# Novel Conjugated *s*-Tetrazine Derivatives Bearing a 4*H*-1,2,4-Triazole Scaffold: Synthesis and Luminescent Properties

**DOI:** 10.3390/molecules27020459

**Published:** 2022-01-11

**Authors:** Anna Maj, Agnieszka Kudelko, Marcin Świątkowski

**Affiliations:** 1 Department of Chemical Organic Technology and Petrochemistry, The Silesian University of Technology, Krzywoustego 4, PL-44100 Gliwice, Poland; anna.Maj@polsl.pl; 2 Institute of General and Ecological Chemistry, Lodz University of Technology, Zeromskiego 116, PL-90924 Lodz, Poland; marcin.swiatkowski@p.lodz.pl

**Keywords:** *s*-tetrazine, 4*H*-1,2,4-triazole, Pinner reaction

## Abstract

A series of new symmetrical *s*-tetrazine derivatives, coupled via a 1,4-phenylene linkage with a 4*H*-1,2,4-triazole ring, were obtained. The combination of these two rings in an extensively coupled system has significant potential applications, mainly in optoelectronics. The methodology used turned out to be useful regardless of the type of five-membered ring or the nature of the individual substituents. All the products were identified by spectroscopic methods, and the target compounds were tested for luminescent properties. This study showed that all the synthesized highly-conjugated triazoles exhibited luminescence; in particular, one derivative, 3,6-bis(4-(5-(4-methoxyphenyl)-4-phenyl-4*H*-1,2,4-triazol-3-yl)phenyl)-1,2,4,5-tetrazine (**13b**), showed strong fluorescence emission and ahigh quantum yield close to 1.

## 1. Introduction

Nitrogen-rich heterocycles are one of the most intriguing groups of compounds in organic chemistry. Among the six-membered systems, 1,2,4,5-tetrazine (*s*-tetrazine) derivatives are of particular interest. Unsubstituted *s*-tetrazine is characterized by having the maximum nitrogen content possible without compromising the stability of the system. Initially, such arrangements were mainly used in the field of high-energy-density materials, because their thermal decomposition leads to ring opening, resulting in the formation of nitriles and nitrogen molecules [1,2,3]. However, in recent years, *s*-tetrazine-containing compounds have also been intensively researched in terms of their applicability in medicine (due to their anti-tuberculosis, anti-cancer, or anti-malaria effects) [4,5,6], bio-orthogonal chemistry (as aconsequence of their high reactivity in Diels-Alder reactions with inverse electron demand) [7,8,9,10] and optoelectronics. Their potential applications in the latter area are strongly related to their characteristic low-energy n→π electronic transitions. This feature can be used in the production of organic light-emitting diodes (OLEDs), organic field-effect transistors (OFETs), or solar cells. The ring in question is also apromising building block in ambipolar and n-type materials, due to its high electron affinity and strongly electron-deficient nature [11,12]. Thus, many scientists are currently working on the synthesis of new *s*-tetrazine structures with superior properties.

Among the many developed procedures for the synthesis of *s*-tetrazine derivatives, one of the most frequently used is the Pinner method and its modifications. This protocol involves the use of carbonitriles and hydrazine hydrate as substrates, as well as an activating agent, such as sulfur. As with all *s*-tetrazine production routes, it is also necessary to oxidize the intermediate dihydro derivative that is formed on the corresponding aromatic system [13,14]. This approach is distinguished above all by the wide range of substrates that can be used. Our previous research shows that it enables, for example, the formation of complex coupled systems that contain additional five-membered rings. So far, we have successfully combined *s*-tetrazine with arange of 1,3,4-oxadiazoles and 1,3,4-thiadiazoles, and the obtained products showed excellent luminescent properties [15,16]. In acontinuation of our research program on the synthesis of *s*-tetrazine derivatives conjugated to five-membered heterocyclic arrangements, we decided to introduce another five-membered ring, 4*H*-1,2,4-triazole.

In fact, 4*H*-1,2,4-triazoles are often described in terms of their biological activity, such as the possession of antiviral, anti-migraine, antifungal, anti-cancer, or psychotropic properties, and some of them are currently used in commercially available products [17,18,19,20]. They are also of great importance in optoelectronics, where their outstanding ability to transport electrons is adirect consequence of the presence of three nitrogen atoms responsible for electron deficiency within the ring, making it an acceptor. Consequently, oligomers containing the 4*H*-1,2,4-triazole ring are extremely popular in the production of blue OLEDs [21,22,23,24]. It is therefore to be expected that coupled systems containing this five-membered ring will show even higher values of fluorescence quantum yield. Due to the importance of 4*H*-1,2,4-triazole derivatives, a wide range of methods for their synthesis can be found in the literature. Most of them are based on the formation of a ring from acyclic derivatives. The most popular substrates include *N,N′*-diacylhydrazines, *N*-cyanoguanidine, isothiocyanates, hydrazides, aminoethylidenehydrazones, aldehydes, and semicarbazides [25].

Searching for the best synthetic methodology, we decided to use the first group of compounds: *N,N′*-diacylhydrazines, because a similar approach was successful in the preparation of analogous systems containing 1,3,4-oxadiazole and 1,3,4-thiadiazole cores. A synthetic pathway designed in such a manner would confirm the versatility of the applied methodology. Moreover, the examination of the luminescence properties of the obtained products makes it possible to analyze the influence of the type of the five-membered ring on fluorescence quantum yield and Stokes shifts. An interesting possibility is the comparison of the results obtained for systems with either an aliphatic or aromatic substituent on the nitrogen atom of the 4*H*-1,2,4-triazole.

## 2. Results and Discussion

### 2.1. Synthesis

**Scheme 1:**
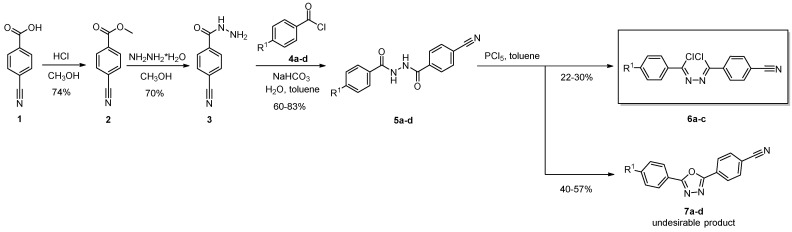
Synthesis of imidoyl chlorides (**6a**–**d**) and 1,3,4-oxadiazoles (**7a**–**d**).

Our previous research showed that the route using diacylhydrazine derivatives is a very favorable pathway for the synthesis of *s*-tetrazine derivatives conjugated via a 1,4-phenylene linker with five-membered rings [15,16]. These compounds have been successfully obtained by a three-step methodology using commercially available 4-cyanobenzoic acid (**1**) as astarting material, which was converted into the corresponding methyl ester (**2**), and then the hydrazide (**3**). The great advantage of this procedure is the possibility of introducing various groups by the reaction with selected acid chlorides (**4a**–**d**). In the case of the previously prepared systems containing 1,3,4-oxadiazole or 1,3,4-thiadiazole cores, diacylhydrazine derivatives (**5a**–**d**) were then cyclized to the appropriate ring under the influence of a suitably selected agent. The synthesis of the 4*H*-1,2,4-triazole ring requires the introduction of an additional step, which is the preparation of imidoyl chlorides (**6a**–**c**, Scheme 1). For this purpose, phosphorus pentachloride is most often used, the reaction being carried out under reflux. At this stage, the formation of an 1,3,4-oxadiazole derivative side-product is a significant complication; therefore, it is extremely important to find the best reaction conditions. In the literature, there are reports of carrying out an analogous transformation (for example the synthesis of 1,2-bis((4-bromophenyl)chloromethylene)hydrazine) for as long as several hours [26]. However, in the case of the compounds in question (**5a**–**d**), after 30 min of heating, the undesired 1,3,4-oxadiazole derivative (**7a**–**d**) was almost exclusively observed. Therefore, attempts were made to limit the ongoing cyclization. Lowering the heating time favorably affected the yield of the desired chlorides, but did not inhibit cyclization, and, in all cases, the 1,3,4-oxadiazole derivatives were still observed. Accordingly, attempts were also made to lower the reaction temperature to room temperature. Unfortunately, they were unsuccessful and as aresult, the reaction did not occur even after the use of ultrasounds. At slightly higher temperatures, the undesired 1,3,4-oxadiazole compound was formed almost exclusively, which was probably aconsequence of the extended concentration process of the mixture on the rotary evaporator, and thus the longer contact of the substrate with phosphorus pentachloride. The transformation was also carried out with another popular agent, oxalyl chloride, in the presence of 2,6-lutidine, but this procedure did not give apositive result. Therefore, we decided to carry out the reaction with phosphorus pentachloride at the boiling point of the solvent, ensuring the shortest possible reaction time. This procedure made it possible to obtain imidoyl chlorides, both those unsubstituted at the phenyl group (R^1^=H, **6a**, Entry 1, Table 1) and those with electron-donating groups (R^1^=OCH_3_, *t*-Bu, **6b**–**c**, Entries 2 and 3, Table 1). However, in the case of the electron-withdrawing nitro group (R^1^=NO_2_, **6d**, Entry 4, Table 1), the reduction in reaction time was not enough to isolate the desired product, and only the 1,3,4-oxadiazole derivative **7d** was observed. We assumed that due to mesomeric effect, the electron-withdrawing NO_2_ substituent situated at para position of the intermediate **5d** increased the polarization of carbonyl group, facilitating the cyclization to 1,3,4-oxadiazole derivative **7d**.

**Scheme 2:**
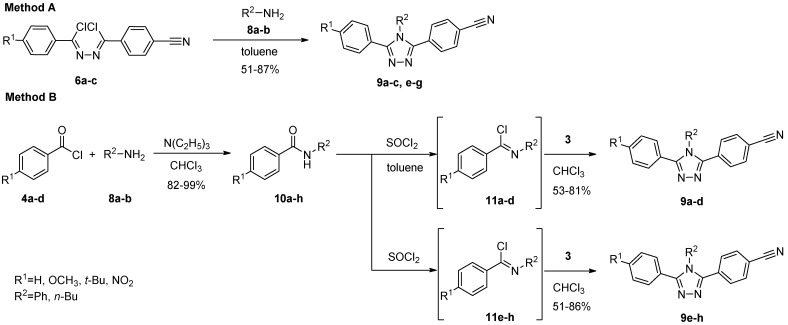
Synthesis of precursors containing a 4*H*-1,2,4-triazole ring (**9a**–**h**).

The resulted imidoyl chlorides **6a**–**c** were directed to the next step—the formation of a 4*H*-1,2,4-triazole ring (Method A, Scheme 2). For this purpose, two amines were used, in order to obtain intermediates 4*H*-1,2,4-triazoles differently substituted at the nitrogen atom (**9a**–**c, e**–**g**). Aniline was chosen as arepresentative aromatic amine (R^2^=Ph), while *n*-butylamine was used as an aliphatic system (R^2^=Bu). In both cases the desired products **9a**–**c, e**–**g** were obtained in satisfactory yields (51–87%, Table 2). Trials to avoid isolation of troublesome imidoyl chlorides **6a**–**d**, and application of one-pot methodology making use of *N,N′*-diacylhydrazine **5a**–**d**, PCl_5_ and subsequent treatment with amines **8a**–**b** did not give better yields; the formation of 1,3,4-oxadiazoles **7a**–**d** was still preferred.

Due to the low yields of the intermediate imidoyl chlorides and the inability to obtain a precursor containing a nitro substituent, an alternative synthesis pathway for systems containing the 4*H*-1,2,4-triazole ring was elaborated (Method B, Scheme 2). In the first step, acid chlorides **4a**–**d** were reacted with amines **8a**–**b** in the presence of triethylamine. The thus obtained amides **10a**–**h** were treated with thionyl chloride, or thionyl chloride in toluene at reflux temperature. The choice of conditions depended on the structure of the substrate, as differences in the efficiency of the reaction were observed due to the individual substituents on the nitrogen atom. The crude chlorides **11a**–**h** were cyclized with the previously prepared hydrazide **3** to give the corresponding 4*H*-1,2,4-triazole derivatives **9a**–**h**. The cyclization step proceeded with similar yield to the previous method (Table 2), however this approach allowed us to avoid a troublesome stage of imidoyl chlorides **6a**–**d** formation, which had a very negative effect on the efficiency of the entire process. Moreover, the methodology turned out to be effective also for systems containing the electron-withdrawing nitro group, which led to an extension of the range of obtained final products.

**Scheme 3:**
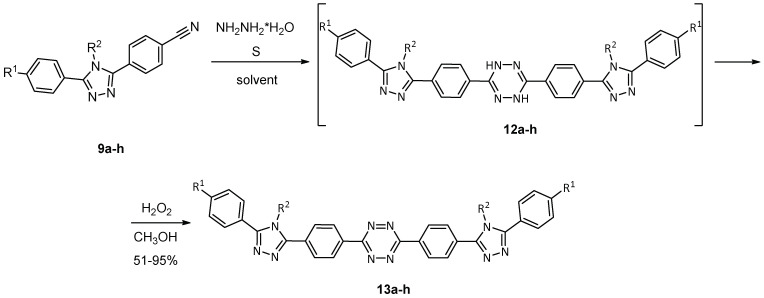
Synthesis of *s*-tetrazine derivatives coupled via a 1,4-phenylene linkage with a 4*H*-1,2,4-triazole ring (**13a**–**h**).

The prepared compounds **9a**–**h** were then used in the Pinner reaction (Scheme 3). Similarly to our previous study, sulfur was used as the activating agent. Optimization of the reaction conditions for 4-(4,5-diphenyl-4*H*-1,2,4-triazol-3-yl)benzonitrile (**9a**), hydrazine hydrate, and sulfur showed that the formation of the *s*-tetrazine core proceeded better with ethanol as the solvent than in non-polar toluene (Entries 1 and 2, Table 3). The last step was to oxidize the obtained dihydro derivatives of *s*-tetrazine (**12a**–**h**) to the corresponding aromatic systems (**13a**–**h**). As in previous studies, hydrogen peroxide was used as the oxidizing agent, and the high yields of products obtained in this way confirmed the versatility of the developed protocol in relation to various five-membered rings. The methodology also worked regardless of the type of substituent on the terminal benzene ring, or on the nitrogen atom in the triazole ring.

The structure of all obtained intermediates and final products was confirmed by ^1^H and ^13^C NMR spectroscopy (Supplementary Materials). The most characteristic and diagnostic signals in ^13^C NMR spectra of **13a**–**h** come from the carbon atoms of the heterocyclic rings: 4*H*-1,2,4-triazole and 1,2,4,5-tetrazine. The highest shifts (low field) correspond to the *s*-tetrazine carbon atom (~169 ppm). Slightly lower shifts were observed for the benzene carbon attached to the methoxy group (above 160 ppm, **13b** and **13f**), the *tert*-butyl group (above 150 ppm, **13c** and **13g**) and the nitro group (above 147 ppm, **13d** and **13h**). The five-membered triazole ring was identified by signals above 150 ppm. Chemical shifts further up the field were typical of aliphatic carbons from the alkyl groups of the butyl chain linked to a triazole ring (13–45 ppm), methoxy (OCH_3_, ~55 ppm) and *tert*-butyl (*t*-Bu, 30–35 ppm) groups adjacent to the terminal benzene ring. 

### 2.2. Luminescent Properties

UV-Vis absorption and 3D fluorescence spectra were recorded for compounds **13a**–**h** (Figures S39–S48 in Supplementary Materials), all of which possessed one emission maximum. The emission wavelengths (λ_em_) fell in the range 374–409 nm (Table 4), which meant that all the compounds exhibited violet fluorescence. In *s*-tetrazine and its derivatives, n→π* transitions are the source of fluorescence [27,28,29]. In the studied compounds, the excitation occurred from orbitals involving tetrazine as well as triazole rings, which was confirmed by the location (λ_ex_ and λ_em_) of the emission maxima in the 3D fluorescence spectra, which were related to the R^1^ and R^2^ substituents (Figure S49). The substituent R^1^ influenced the λ_em_, whereas R^2^ influenced the λ_ex_. The stronger the electron-donating group (EDG) as R^1^, the larger was the induced bathochromic shift of λ_em_. Therefore, λ_em_ increased as follows: H < *t*-Bu < OCH_3_, in accordance with the previously reported analogous *s*-tetrazine-1,3,4-oxadiazole derivatives [15]. On the other hand, the compounds with *n*-Bu as R^2^ gave a hypsochromic shift of λ_ex_ in comparison to the Ph substituent, which was a weaker EDG. In consequence, the Stokes shift of the *n*-Bu compounds was larger than the respective Ph compounds. The R^1^ and R^2^ groups also gave different quantum yields (Φ). For compounds **13a**–**c** (R^2^=Ph), the quantum yield depended strongly on R^1^, whereas for **13e**–**g** (R^1^=*n*-Bu), the quantum yields were comparable (Table 4). This trend indicated that in the case of **13e**–**g**, fluorescence occurred from the excited state, in which most of the electron density was localized outside the phenyl rings with R^1^ substituents. Compounds **13d** and **13h** possessing NO_2_ group as R^1^ did not follow above relationships. The NO_2_ group is an electron-withdrawing group (EWG), which, in contrast to EDG, leads to a decrease of electron density in a fluorophore moiety. As a result, the fluorescence was almost completely quenched in the case of **13d** and **13h**. A significant decrease of a fluorescence intensity and consequently a quantum yield was also observed for analogous *s*-tetrazine compounds with NO_2_ groups [15,16]. The most efficient fluorescent compound in the studied group was **13b**. It exhibited both the strongest fluorescence and aquantum yield close to 1. Its properties were exceptional in comparison to similar *s*-tetrazine derivatives. Such high quantum yield values are possessed by *s*-tetrazines directly conjugated to oxadiazole or thiadiazole rings [30]. Compounds analogous to the studied ones, in which heteroatomic rings are separated by phenyl rings, typically achieve quantum yields no higher than 0.60 [15,16]. Two factors may be responsible for the phenomenon of **13b**. Firstly, the conjugation of the molecular orbitals of the tetrazine ring with triazole rings (in **13b**) is more effective than with oxadiazole [15] or thiadiazole [16] rings, due to the same heteroatoms being present. Secondly, each triazole ring in **13b** is bonded to three benzene rings. As a consequence, the distribution of electron density within a fluorophore moiety is different in comparison to oxadiazole and thiadiazole analogs, in which five-memebred rings are connected only to two benzene rings [15,16].

## 3. Experimental

### 3.1. General Information

All reagents were purchased from commercial sources and used without further purification. Melting points were measured on a Stuart SMP3 melting point apparatus (Stuart, Staffordshire, UK). NMR spectra were recorded at 25 °C on an Agilent 400-NMR spectrometer (Agilent Technologies, Waldbronn, Germany) at 400 MHz for ^1^H and 100 MHz for ^13^C, using CDCl_3_ or DMSO as solvent and TMS as the internal standard. UV-Vis absorption and 3D fluorescence spectra were registered in methanol or dichloromethane solutions (c = 5 × 10^−6^ mol/dm^3^) with Jasco V-660 and Jasco F-6300 spectrometers (Jasco Corporation, Tokyo, Japan), respectively. FT-IR spectra were measured between 4000 and 650 cm^−1^ on an FT-IR Nicolet 6700 apparatus (Thermo Fischer Scientific, Wesel, Germany) with a Smart iTR accessory. Elemental analyses were performed with a VarioELanalyser (Elementar UK Ltd., Stockport, UK). High-resolution mass spectra were obtained by means of a Waters ACQUITY UPLC/Xevo G2QT instrument (Waters Corporation, Milford, MA, USA). Thin-layer chromatography was performed on silica gel 60 F254 (Merck KGaA, Darmstadt, Germany) thin-layer chromatography plates using chloroform, chloroform/ethyl acetate (1:1 *v*/*v*) or chloroform/ethyl acetate (5:1 *v*/*v*) as the mobile phases.

### 3.2. Synthesis and Characterization

Compounds **1**–**5** were synthesized according to the literature [15].

#### 3.2.1. Synthesis of Imidoyl Chlorides (**6a**–**c**)

The crushed starting material (**5a**–**c**, 1.5 mmol) was suspended in toluene (40 mL) and brought to boiling. Phosphorus pentachloride (3.0 mmol) was added to the hot solution and the mixture was immediately concentrated on arotary evaporator. The residue was dissolved in chloroform (40 mL) and washed with distilled water (2 × 40 mL). The organic layer was dried and evaporated on arotary evaporator. The crude product was purified by column chromatography using chloroform as the mobile phase to give the corresponding purified chloride (**6a**–**c**).

*N-chloro(phenyl)methylidene-4-cyanobenzene-1-carbohydrazonoyl chloride* (**6a**). The product was obtained asyellow powder (0.14 g, 30%); mp 132–133 °C. UV (CH_3_OH) λ_max_ (log ε) 204 (4.35), 278 (4.29) nm; IR (ATR) ν_max_ 3091, 3061, 2227, 1981, 1942, 1594, 1558, 1497, 1487, 1445, 1404, 1307, 1292, 1224, 1177, 1115, 1076, 1018, 1000, 979, 919, 847, 808, 759, 712, 684, 672, 642, 620, 611, 596, 581 cm^–1^; ^1^H-NMR (400 MHz, CDCl_3_): δ 7.47–7.50 (m, 2H, Ar), 7.51–7.56 (m, 1H, Ar), 7.77 (d, 2H, *J* = 8.0 Hz, Ar), 8.12–8.14 (m, 2H, Ar), 8.24 (d, 2H, *J* = 8.0 Hz, Ar); ^13^C-NMR (100 MHz, CDCl_3_): δ 115.3, 118.2, 128.8, 129.1, 131.6, 132.3, 132.4, 133.4, 137.7, 142.3, 145.3. Anal. calc. for C_15_H_9_Cl_2_N_3_: C, 59.63; H, 3.00; N, 13.91. Found: C, 59.60; H, 3.04; N, 13.96; HRMS (ESI): *m*/*z* calcd for C_15_H_9_Cl_2_N_3_+H^+^: 302.0252; found: 302.0253.
*N-chloro(4-methoxyphenyl)methylidene-4-cyanobenzene-1-carbohydrazonoyl chloride* (**6b**). The product was obtained as yellow powder (0.11 g, 22%); mp 123–124 °C. UV (CH_3_OH) λ_max_ (log ε) 202.5 (4.13), 255 (3.86), 292.5 (4.05), 328 (3.95) nm; IR (ATR) ν_max_ 3096, 2995, 2839, 2572, 2223, 2051, 1942, 1596, 1667, 1505, 1464, 1441, 1418, 1405, 1305, 1252, 1229, 1169, 1118, 1032, 918, 842, 831, 808, 790, 767, 724, 648, 635, 618, 602, 584 cm^–1^; ^1^H-NMR (400 MHz, CDCl_3_): δ 3.89 (s, 3H, OCH_3_), 7.47 (d, 2H, *J* = 12.0 Hz, Ar), 7.76 (d, 2H, *J* = 8.0 Hz, Ar), 8.10 (d, 2H, *J* = 12.0 Hz, Ar) 8.24 (d, 2H, *J* = 8.0 Hz, Ar); ^13^C-NMR (100 MHz, CDCl_3_): δ 55.7, 114.1, 115.1, 118.2, 125.9, 129.1, 130.7, 132.4, 137.9, 142.6, 146.1, 163.1. Anal. calc. for C_16_H_11_Cl_2_N_3_O: C, 57.85; H, 3.34; N, 12.65. Found: C, 57.88; H, 3.32; N, 12.69; HRMS (ESI): *m*/*z* calcd for C_16_H_11_Cl_2_N_3_O+H^+^: 332.0357; found: 332.0356.
*4-(tert-butyl)-N-(chloro(4-cyanophenyl)methylene)benzohydrazonoyl chloride* (**6c**). The product was obtained as yellow powder (0.14 g, 26%); mp 163–164 °C. UV (CH_3_OH) λ_max_ (log ε) 203 (4.07), 282.5 (4.05) nm; IR (ATR) ν_max_ 3092, 2970, 2227, 1934, 1590, 1552, 1499, 1475, 1403, 1368, 1269, 1229, 1193, 1177, 1111, 1016, 922, 850, 838, 802, 768, 724, 712, 648, 636, 618, 601, 581 cm^–1^; ^1^H-NMR (400 MHz, CDCl_3_): δ 1.37 (s, 9H, C(CH_3_)_3_), 7.51 (d, 2H, *J* = 8.0 Hz, Ar), 7.77 (d, 2H, *J* = 8.0 Hz, Ar), 8.06 (d, 2H, *J* = 8.0 Hz, Ar) 8.24 (d, 2H, *J* = 8Hz, Ar); ^13^C-NMR (100 MHz, CDCl_3_): δ 31.3, 35.2, 115.2, 118.2, 125.8, 128.7, 129.1, 130.7, 132.4, 137.8, 142.3, 145.5, 156.2. Anal. calc. for C_19_H_17_Cl_2_N_3_: C, 63.70; H, 4.78; N, 11.73. Found: C, 63.74; H, 4.75; N, 11.71; HRMS (ESI): *m*/*z* calcd for C_19_H_17_Cl_2_N_3_+H^+^: 358.0878; found: 358.0876.

#### 3.2.2. Synthesis of 4-(5-Phenyl-4*H*-1,2,4-triazol-3-yl)benzonitrile Derivatives (**9a**–**h**)

Method A: The substrate (**6a**–**c**, 2.0 mmol) was dissolved in toluene (50 mL) and cooled to 0 °C. The appropriate amine (8.0 mmol) was added dropwise and the mixture was stirred in an ice bath for 3 h. It was then allowed to reach room temperature and was stirred for 24 h. Next, it was heated under reflux for another 24 h and evaporated on arotary evaporator. The residue was washed with small amount of cold ethanol to give pure product (**9a**–**c, e**–**g**).

Method B: The amides **10a**–**h** and chlorides **11a**–**h** were obtained according to the methodologies described in the literature [34,35,36]. The crude imidoyl chloride (**11a**–**h**, 2.2 mmol) and 4-cyanobenzohydrazide (**3**, 2.0 mmol) were dissolved in chloroform (10 mL) and heated under reflux for 48 h. The mixture was then cooled to room temperature, filtered and evaporated on rotary evaporator. The residue was washed with small amount of cold ethanol to give pure product (**9a**–**h**).

4*-(4,5-diphenyl-4H-1,2,4-triazol-3-yl)benzonitrile* (**9a**). The product was obtained as white powder method A: (0.50 g, 78%, method B: 0.52 g, 81%); mp 227–228 °C (li. 225–227 °C [37]). UV (CH_3_OH) λ_max_ (log ε) 204 (4.63), 270.5 (4.34) nm; IR (ATR) ν_max_ 3052, 2557, 2232, 1609, 1494, 1467, 1446, 1427, 1276, 1181, 1151, 1078, 1019, 973, 848, 790, 772, 752, 739, 714, 696, 685, 645, 623, 611, 567 cm^–1^; ^1^H-NMR (400 MHz, DMSO-*d*_6_): δ 7.33–7.35 (m, 2H, Ar), 7.36–7.38 (m, 1H, Ar), 7.40 (d, 2H, *J* = 8.0 Hz, Ar), 7.41–7.42 (m, 1H, Ar) 7.46–7.47 (m, 2H, Ar), 7.48–7.51 (m, 2H, Ar) 7.57 (d, 2H, *J* = 12.0 Hz, Ar), 7.85 (d, 2H, *J* = 8.0 Hz, Ar); ^13^C-NMR (100 MHz, DMSO-*d*_6_): δ 112.2, 118.2, 128.2, 128.5, 128.5, 129.0, 129.8, 130.0, 130.1, 131.3, 132.4, 134.4, 152.9, 154.8. Anal. calc. for C_21_H_14_N_4_: C, 78.24; H, 4.38; N, 17.38. Found: C, 78.27; H, 4.36; N, 17.33; HRMS (ESI): *m*/*z* calcd for C_21_H_14_N_4_+H^+^: 323.1297; found: 323.1299.
*4-[5-(4-methoxyphenyl)-4-phenyl-4H-1,2,4-triazol-3-yl]benzonitrile* (**9b**). The product was obtained as white powder (method A: 0.53 g, 75%, method B: 0.54 g, 77%); mp 223–224 °C. UV (CH_3_OH) λ_max_ (log ε) 203 (4.58), 238.5 (4.19), 281 (4.20) nm; IR (ATR) ν_max_ 2813, 2588, 2226, 2011, 1609, 1577, 1533, 1495, 1471, 1461, 1435, 1412, 1336, 1304, 1287, 1254, 1177, 1109, 1044, 1021, 992, 971, 849, 832, 806, 784, 768, 740, 708, 697, 687, 661, 625, 614, 592, 578, 571 cm^–1^; ^1^H-NMR (400 MHz, CDCl_3_): δ 3.79 (s, 3H, OCH_3_), 6.81 (d, 2H, *J* = 8.0 Hz, Ar), 7.18 (d, 2H, *J*= 8.0 Hz, Ar), 7.35 (d, 2H, *J* = 8.0 Hz, Ar) 7.47–7.51 (m, 3H, Ar), 7.54 (d, 2H, *J* = 12.0 Hz, Ar), 7.58 (d, 2H, *J* = 12.0 Hz, Ar); ^13^C-NMR (100 MHz, CDCl_3_): δ 55.4, 113.3, 114.2, 118.3, 118.7, 127.9, 129.1, 130.3, 130.4, 130.5, 131.5, 132.3, 135.0, 152.9, 155.5, 161.0. Anal. calc. for C_22_H_16_N_4_O: C, 74.98; H, 4.58; N, 15.90. Found: C, 74.97; H, 4.55; N, 15.94; HRMS (ESI): *m*/*z* calcd for C_22_H_16_N_4_O+H^+^: 353.1402; found: 353.1401.
*4-[5-(4-tert-butylphenyl)-4-phenyl-4H-1,2,4-triazol-3-yl]benzonitrile* (**9c**). The product was obtained as yellow powder (method A: 0.39 g, 51%, method B: 0.44 g, 58%); mp 160–161 °C. UV (CH_3_OH) λ_max_ (log ε) 203.5 (4.58), 283.5 (4.56) nm; IR (ATR) ν_max_ 3093, 2971, 2592, 2226, 2161, 1997, 1932, 1590, 1551, 1499, 1475, 1403, 1368, 1308, 1289, 1269, 1229, 1193, 1177, 1111, 1016, 921, 850, 837, 768, 724, 713, 649, 636, 619, 602, 592, 582, 571 cm^–1^; ^1^H-NMR (400 MHz, CDCl_3_): δ 1.28 (s, 9H, C((CH_3_)_3_), 6.68–6.71 (m, 2H, Ar), 6.76 (t, 1H, *J* = 8.0 Hz, Ar), 7.31 (d, 2H, *J* = 12.0 Hz, Ar) 7.36 (d, 2H, *J* = 12.0 Hz, Ar), 7.50 (d, 2H, *J* = 8.0 Hz, Ar), 7.55 (d, 2H, *J* = 8.0 Hz, Ar), 7.57 (d, 2H, *J* = 4.0 Hz, Ar); ^13^C-NMR (100 MHz, CDCl_3_): δ 31.2, 34.9, 113.3, 115.4, 118.3, 118.8, 123.6, 125.6, 127.9, 128.5, 129.1, 130.5, 132.3, 135.1, 153.0, 153.4, 155.6. Anal. calc. for C_25_H_22_N_4_: C, 79.34; H, 5.86; N, 14.80. Found: C, 79.37; H, 5.81; N, 14.82; HRMS (ESI): *m*/*z* calcd for C_25_H_22_N_4_+H^+^: 379.1923; found: 379.1925.
*4-[5-(4-nitrophenyl)-4-phenyl-4H-1,2,4-triazol-3-yl]benzonitrile* (**9d**). The product was obtained as yellow powder (method B: 0.39 g, 53%); mp 221–222 °C. UV (CH_3_OH) λ_max_ (log ε) 212 (4.52), 293 (4.51) nm; IR (ATR) ν_max_ 3428, 3277, 3061, 2960, 2931, 2860, 2228, 2149, 1980, 1947, 1641, 1602, 1575, 1549, 1489, 1447, 1413, 1316, 1284, 1273, 1176, 1154, 1105, 1077, 1014, 999, 964, 922, 854, 802, 775, 739, 708, 687 cm^–1^; ^1^H-NMR (400 MHz, DMSO-*d*_6_): δ 7.58–7.61 (m, 5H, Ar), 7.64 (d, 2H, *J* = 8.0 Hz, Ar), 7.93 (d, 2H, *J* = 8.0 Hz, Ar), 8.28 (d, 2H, *J* = 8.0 Hz, Ar); ^13^C-NMR (100 MHz, DMSO-*d*_6_): δ 112.3, 118.0, 123.6, 128.0, 129.0, 129.5, 130.1, 130.3, 130.9, 132.3, 132.6, 133.8, 147.8, 153.1, 153.5. Anal. calc. for C_21_H_13_N_5_O_2_: C, 68.66; H, 3.57; N, 19.06. Found: C, 68.68; H, 3.54; N, 19.09; HRMS (ESI): *m*/*z* calcdfor C_21_H_13_N_5_O_2_+H^+^: 368.1147; found: 368.1145.
*4-(4-butyl-5-phenyl-4H-1,2,4-triazol-3-yl)benzonitrile* (**9e**). The product was obtained as white powder (method A: 0.32 g, 53%, method B: 0.33 g, 55%); mp 166–167 °C. UV (CH_3_OH) λ_max_ (log ε) 204 (4.70), 261 (4.52) nm; IR (ATR) ν_max_ 3069, 2959, 2926, 2860, 2318, 2234, 2162, 2027, 1981, 1614, 1473, 1464, 1443, 1419, 1397, 1356, 1327, 1280, 1248, 1158, 1112, 1083, 1074, 1033, 1022, 971, 929, 883, 858, 831, 858, 831, 744, 731, 698, 659, 645, 617, 591 cm^−1^; ^1^H-NMR (400 MHz, CDCl_3_): δ 0.65 (t, 3H, *J* = 8.0 Hz, CH_3_), 1.01 (sextet, 2H, *J* = 8.0 Hz, CH_2_), 1.36 (quintet, 2H, *J* = 8.0 Hz, CH_2_), 4.12 (t, 2H, *J* = 8.0 Hz, CH_2_), 7.53–7.56 (m, 3H, Ar), 7.65–7.67 (m, 2H, Ar), 7.82–7.87 (m, 4H, Ar); ^13^C-NMR (100 MHz, CDCl_3_): δ 13.2, 19.3, 32.1, 45.0, 114.0, 118.2, 127.4, 129.1, 129.2, 129.5, 130.5, 132.5, 132.8, 153.8, 156.5. Anal. calc. for C_19_H_18_N_4_: C, 75.47; H, 6.00; N, 18.53. Found: C, 75.44; H, 6.05; N, 18.54; HRMS (ESI): *m*/*z* calcd for C_19_H_18_N_4_+H^+^: 303.1610; found: 303.1611.
*4-[4-butyl-5-(4-methoxyphenyl)-4H-1,2,4-triazol-3-yl]benzonitrile* (**9f**). The product was obtained as yellow powder (method A: 0.49 g, 76%, method B: 0.48 g, 75%); mp 141–142 °C. UV (CH_3_OH) λ_max_ (log ε) 202.5 (4.48), 237.5 (4.23), 269 (4.21) nm; IR (ATR) ν_max_ 2965, 2936, 2875, 2547, 2229, 2179, 2032, 1937, 1612, 1576, 1538, 1480, 1470, 1426, 1414, 1393, 1377, 1362, 1340, 1296, 1254, 1172, 1111, 1094, 1031, 971, 942, 886, 835, 822, 800, 781, 756, 744, 725, 671, 634, 624, 588, 582 cm^−1^; ^1^H-NMR (400 MHz, CDCl_3_): δ 0.65 (t, 3H, *J* = 8.0 Hz, CH_3_), 1.01 (sextet, 2H, *J* = 8.0 Hz, CH_2_), 1.36 (quintet, 2H, *J* = 8.0 Hz, CH_2_), 3.87 (s, 3H, OCH_3_), 4.09 (t, 2H, *J* = 8.0 Hz, CH_2_), 7.04 (d, 2H, *J* = 8.0 Hz, Ar), 7.58 (d, 2H, *J* = 8.0 Hz, Ar), 7.80–7.87 (m, 4H, Ar); ^13^C-NMR (100 MHz, CDCl_3_): δ 13.3, 19.4, 32.0, 45.1, 55.6, 114.0, 114.7, 118.2, 119.1, 129.5, 130.6, 132.2, 132.9, 153.6, 156.2, 161.4. Anal. calc. for C_20_H_20_N_4_O: C, 72.27; H, 6.06; N, 16.86. Found: C, 72.24; H, 6.08; N, 16.82; HRMS (ESI): *m*/*z* calcd for C_20_H_20_N_4_O+H^+^: 333.1715; found: 333.1714.
*4-[4-butyl-5-(4-tert-butylphenyl)-4H-1,2,4-triazol-3-yl]benzonitrile* (**9g**). The product was obtained as white powder (method A: 0.62 g, 87%, method B: 0.61 g, 85%); mp 193–194 °C. UV (CH_3_OH) λ_max_ (log ε) 203 (4.56), 233.5 (4.27), 263.5 (4.32) nm; IR (ATR) ν_max_ 2960, 2929, 2872, 2228, 1612, 1562, 1472, 1427, 1394, 1359, 1278, 1267, 1200, 1171, 1117, 1018, 972, 924, 857, 846, 833, 772, 754, 746, 726, 673, 634, 610, 599, 588 cm^−1^; ^1^H-NMR (400 MHz, CDCl_3_): δ 0.65 (t, 3H, *J* = 8.0 Hz, CH_3_), 1.01 (sextet, 2H, *J* = 8.0 Hz, CH_2_), 1.32–1.41 (m, 11H,CH_2_, C((CH_3_)_3_), 4.11 (t, 2H, *J* = 8.0 Hz, CH_2_), 7.54 (d, 2H, *J* = 8.0 Hz, Ar) 7.58 (d, 2H, *J* = 12.0 Hz, Ar), 7.81–7.86 (m, 4H, Ar); ^13^C-NMR (100 MHz, CDCl_3_): δ 13.3, 19.4, 31.3, 32.2, 45.0, 113.9, 118.2, 124.3, 126.1, 128.8, 129.5, 132.6, 132.8, 153.7, 153.8, 156.6. Anal. calc. for C_23_H_26_N_4_: C, 77.06; H, 7.31; N, 15.63. Found: C, 77.01; H, 7.33; N, 15.65; HRMS (ESI): *m*/*z* calcd for C_23_H_26_N_4_+H^+^: 359.2236; found: 359.2234.
*4-[4-butyl-5-(4-nitrophenyl)-4H-1,2,4-triazol-3-yl]benzonitrile* (**9h**). The product was obtained as orange powder (method B: 0.35 g, 51%); mp 180–181 °C. UV (CH_3_OH) λ_max_ (log ε) 208 (4.45), 280 (4.46) nm; IR (ATR) ν_max_ 2933, 2872, 2236, 1641, 1603, 1518, 1474, 1394, 1347, 1314, 1290, 1277, 1182, 1108, 1013, 974, 862, 854, 840, 763, 746, 722, 695 cm^−1^; ^1^H-NMR (400 MHz, CDCl_3_): δ 0.68 (t, 3H, *J* = 8.0 Hz, CH_3_) 1.03 (sextet, 2H, *J*=8.0 Hz, CH_2_), 1.39 (quintet, 2H, *J* = 8.0 Hz, CH_2_), 4.17 (t, 2H, *J* = 7.5 Hz, CH_2_), 7.84–7.89 (m, 4H, Ar) 7.92 (d, 2H, *J* = 8.0 Hz, Ar), 8.42 (d, 2H, *J* = 8.0 Hz, Ar); ^13^C-NMR (100 MHz, CDCl_3_): δ 13.1, 19.3, 32.2, 45.3, 114.4, 117.9, 124.3, 129.5, 129.9, 131.6, 132.9, 133.5, 148.9, 154.2, 154.6. Anal. calc. for C_19_H_17_N_5_O_2_: C, 65.69; H, 4.93; N, 20.16. Found: C, 65.67; H, 4.94; N, 20.14; HRMS (ESI): *m*/*z* calcd for C_19_H_17_N_5_O_2_+H^+^: 348.1461; found: 348.1463.

#### 3.2.3. Synthesis of Title Compounds (**13a**–**h**)

The substrate (**9a**–**h**, 1.5 mmol) and sulfur (0.033 g) were suspended in ethanol (60 mL) and hydrazine hydrate (hydrazine 64%, 0.3 mL) was added dropwise. It was heated under reflux for 2 h, then filtered and evaporated on a rotary evaporator. The obtained crude intermediate (**12a**–**h**) was dissolved in methanol (20 mL), hydrogen peroxide was added (hydrogen peroxide solution 34.5–36.5%, 22 mL) and the mixture was stirred at room temperature for 24 h. The resulting mixture was filtered and concentrated on arotary evaporator. Crude product (**13a**–**h**) was purified by column chromatography using chloroform/ethyl acetate (1:1 *v*/*v*) as the mobile phases.

*3,6-bis(4-(4,5-diphenyl-4H-1,2,4-triazol-3-yl)phenyl)-1,2,4,5-tetrazine* (**13a**). The product was obtained as white powder (0.64 g, 63%); mp 235–236 °C. UV (CH_2_Cl_2_) λ_max_ (log ε) 282 (4.59) nm; IR (ATR) ν_max_ 3498, 2232, 1685, 1610, 1564, 1493, 1471, 1445, 1427, 1279, 1183, 1170, 1157, 1076, 1019, 1002, 981, 850, 789, 774, 740, 715, 695, 646, 622, 610, 598, 584 cm^−1^; ^1^H-NMR (400 MHz, DMSO-d_6_): δ 7.38–7.42 (m, 10H, Ar), 7.47–7.51 (m, 10H, Ar), 7.57 (d, 4H, *J* = 8.0 Hz, Ar), 7.85 (d, 4H, *J* = 8.0 Hz, Ar); ^13^C-NMR (100 MHz, DMSO-d_6_): δ 112.2, 118.2, 126.7, 127.5, 128.2, 128.3, 128.5, 129.0, 130.0, 131.4, 132.4, 134.4, 152.9, 154.9, 167.1. Anal. calc. for C_42_H_28_N_10_: C, 74.98; H, 4.20; N, 20.82. Found: C, 74. 95; H, 4.22; N, 20.85; HRMS (ESI): *m*/*z* calcd for C_42_H_28_N_10_+H^+^: 673.2577; found: 673.2576.
*3,6-bis(4-(5-(4-methoxyphenyl)-4-phenyl-4H-1,2,4-triazol-3-yl)phenyl)-1,2,4,5-tetrazine* (**13b**). The product was obtained as white powder (1.04 g, 95%); mp 219–220 °C. UV (CH_2_Cl_2_) λ_max_ (log ε) 289 (4.65) nm; IR (ATR) ν_max_ 3449, 2975, 2938, 2837, 2233, 2161, 2028, 1684, 1606, 1576, 1533, 1494, 1472, 1454, 1433, 1416, 1335, 1306, 1289, 1257, 1191, 1178, 1110, 1074, 1021, 979, 922, 847, 832, 808, 785, 748, 700, 657, 635, 609, 599, 592, 578 cm^−1^; ^1^H-NMR (400 MHz, CDCl_3_): δ 3.79 (s, 6H, OCH_3_), 6.81 (d, 4H, *J*=8.0 Hz, Ar), 7.18 (d, 4H, *J* = 8.0 Hz, Ar), 7.34 (d, 4H, *J* = 8.0 Hz, Ar) 7.47−7.59 (m, 14H, Ar); ^13^C-NMR (100 MHz, CDCl_3_): δ 55.4, 114.2, 118.3, 118.8, 127.8, 129.1, 130.3, 130.4, 130.5, 131.5, 132.3, 135.0, 152.9, 155.5, 161.0, 168.6. Anal. calc. for C_44_H_32_N_10_O_2_: C, 72.12; H, 4.40; N, 19.11. Found: C, 72.11; H, 4.44; N, 19.13; HRMS (ESI): *m*/*z* calcd for C_44_H_32_N_10_O_2_+H^+^: 733.2788; found: 733.2789.
*3,6-bis(4-(5-(4-(tert-butyl)phenyl)-4-phenyl-4H-1,2,4-triazol-3-yl)phenyl)-1,2,4,5-tetrazine* (**13c**). The product was obtained as pink powder (0.86 g, 73%); mp 157–158 °C. UV (CH_2_Cl_2_) λ_max_ (log ε) 264 (4.76) nm; IR (ATR) ν_max_ 3359, 3268, 2960, 2868, 2225, 2155, 1609, 1430, 1394, 1350, 1268, 1220, 1202, 1116, 1063, 1014, 979, 967, 925, 837, 772, 751, 742, 698, 659, 624, 598, 591, 581, 572 cm^−1^; ^1^H-NMR (400 MHz, CDCl_3_): δ 1.28 (s, 18H, C((CH_3_)_3_), 7.18–7.21 (m, 4H, Ar), 7.31 (d, 4H, *J* = 8.0 Hz, Ar), 7.35 (d, 4H, *J* = 8.0 Hz, Ar) 7.45–7.59 (m, 14H, Ar); ^13^C-NMR (100 MHz, CDCl_3_): δ 31.2, 34.9, 113.3, 118.3, 123.5, 125.7, 127.9, 128.5, 129.1, 130.3, 130.5, 132.3, 135.0, 153.0, 153.5, 156.2, 168.7. Anal. calc. for C_50_H_44_N_10_: C, 76.51; H, 5.65; N, 17.84. Found: C, 76.54; H, 5.63; N, 17.82; HRMS (ESI): *m*/*z* calcd for C_50_H_44_N_10_+H^+^: 785.3829; found: 785.3826.
*3,6-bis(4-(5-(4-nitrophenyl)-4-phenyl-4H-1,2,4-triazol-3-yl)phenyl)-1,2,4,5-*tetrazine (**13d**). The product was obtained as yellow powder (0.63 g, 55%); mp 234–235 °C. UV (CH_2_Cl_2_) λ_max_ (log ε) 304 (4.67) nm; IR (ATR) ν_max_ 3075, 2960, 2618, 2227, 2169, 1679, 1603, 1514, 1493, 1476, 1455, 1433, 1406, 1346, 1336, 1317, 1289, 1271, 1262, 1155, 1105, 1078, 1015, 973, 922, 863, 845, 802, 786, 773, 760, 741, 724, 699, 686 cm^−1^; ^1^H-NMR (400 MHz, CDCl_3_): δ 7.22 (d, 4H, *J* = 8.0 Hz, Ar), 7.50–7.57 (m, 8H, Ar), 7.60–7.63 (m, 10H, Ar), 8.17 (d, 4H, *J* = 12.0 Hz, Ar); ^13^C-NMR (100 MHz, CDCl_3_): δ 113.8, 117.9, 123.8, 127.5, 129.0, 129.4, 130.6, 130.8, 130.9, 132.3, 134.2, 148.4, 153.5, 153.8, 166.1. Anal. calc. for C_42_H_26_N_12_O_4_: C, 66.14; H, 3.44; N, 22.04. Found: C, 66.15; H, 3.47; N, 22.02; HRMS (ESI): *m*/*z* calcd for C_42_H_26_N_12_O_4_+H^+^: 763.2278; found: 763.2279.
*3,6-bis(4-(4-butyl-5-phenyl-4H-1,2,4-triazol-3-yl)phenyl)-1,2,4,5-tetrazine* (**13e**). The product was obtained as white powder (0.61 g, 64%); mp 161–162 °C. UV (CH_2_Cl_2_) λ_max_ (log ε) 271 (4.57) nm; IR (ATR) ν_max_ 3072, 2958, 2926, 2874, 2233, 2161, 1980, 1691, 1615, 1523, 1474, 1443, 1419, 1395, 1358, 1280, 1248, 1158, 1113, 1094, 1074, 1033, 1022, 971, 930, 858, 832, 777, 744, 731, 699, 659, 643, 617, 591, 585 cm^−1^; ^1^H-NMR (400 MHz, CDCl_3_): δ 0.65 (t, 6H, *J* = 8.0 Hz, CH_3_), 1.01 (sextet, 4H, *J* = 8.0 Hz, CH_2_), 1.36 (quintet, 4H, *J* = 8.0 Hz, CH_2_), 4.12 (t, 4H, *J* = 8.0 Hz, CH_2_), 7.53–7.56 (m, 6H, Ar), 7.65–7.66 (m, 4H, Ar), 7.82–7.87 (m, 8H, Ar); ^13^C-NMR (100 MHz, CDCl_3_): δ 13.2, 19.4, 32.1, 45.1, 114.1, 118.2, 127.2, 129.1, 129.2, 129.5, 130.6, 132.9, 153.8, 156.5, 168.7. Anal. calc. for C_38_H_36_N_10_: C, 72.13; H, 5.73; N, 22.14. Found: C, 72.10; H, 5.75; N, 22.17; HRMS (ESI): *m*/*z* calcd for C_38_H_36_N_10_+H^+^: 633.3203; found: 633.3201.
*3,6-bis(4-(4-butyl-5-(4-methoxyphenyl)-4H-1,2,4-triazol-3-yl)phenyl)-1,2,4,5-tetrazine* (**13f**). The product was obtained as yellow powder (0.83 g, 80%); mp 193–194 °C. UV (CH_2_Cl_2_) λ_max_ (log ε) 273 (4.49) nm; IR (ATR) ν_max_ 3317, 3158, 2971, 2928, 2861, 2274, 1680, 1614, 1579, 1566, 1533, 1483, 1463, 1432, 1381, 1308, 1290, 1254, 1182, 1149, 1111, 1035, 1022, 984, 867, 834, 787, 741, 726, 671, 629, 617, 604, 583 cm^−1^; ^1^H-NMR (400 MHz, CDCl_3_): δ 0.62 (t, 6H, *J* = 8.0 Hz, CH_3_), 0.97 (sextet, 4H, *J* = 8.0 Hz, CH_2_), 1.31 (quintet, 4H, *J* = 8.0 Hz, CH_2_), 3.86 (s, 6H, OCH_3_), 4.05 (t, 4H, *J* = 8.0 Hz, CH_2_), 7.03 (d, 4H, *J* = 8.0 Hz, Ar), 7.57 (d, 4H, *J* = 8.0 Hz, Ar), 7.66 (d, 4H, *J* = 8.0 Hz, Ar), 7.95 (d, 4H, *J* = 8.0 Hz, Ar); ^13^C-NMR (100 MHz, CDCl_3_): δ 13.3, 19.4, 32.1, 45.0, 55.6, 114.7, 119.6, 128.3, 129.1, 130.5, 131.0, 135.2, 154.6, 156.0, 161.3, 168.9. Anal. calc. for C_40_H_40_N_10_O_2_: C, 69.34; H, 5.82; N, 20.22. Found: C, 69.35; H, 5.86; N, 20.20; HRMS (ESI): *m*/*z* calcd for C_40_H_40_N_10_O_2_+H^+^: 693.3414; found: 693.3415.
*3,6-bis(4-(4-butyl-5-(4-(tert-butyl)phenyl)-4H-1,2,4-triazol-3-yl)phenyl)-1,2,4,5-tetrazine* (**13g**). The product was obtained as yellow powder (0.75 g, 67%); mp 71–72 °C. UV (CH_2_Cl_2_) λ_max_ (log ε) 267 (4.36) nm; IR (ATR) ν_max_ 3192, 2962, 2872, 2231, 1671, 1617, 1567, 1481, 1463, 1429, 1395, 1363, 1269, 1220, 1116, 1018, 984, 841, 773, 731, 659, 599, 591, 582 cm^−1^; ^1^H-NMR (400 MHz, CDCl_3_): δ 0.65 (t, 6H, *J* = 8.0 Hz, CH_3_), 1.01 (sextet, 4H, *J* = 8.0 Hz, CH_2_), 1.32–1.36 (m, 22H, CH_2_, C((CH_3_)_3_), 4.11 (t, 4H, *J* = 8.0 Hz, CH_2_), 7.54 (d, 4H, *J* = 8.0 Hz, Ar) 7.58 (d, 4H, *J* = 12.0 Hz, Ar), 7.81–7.86 (m, 8H, Ar); ^13^C-NMR (100 MHz, CDCl_3_): δ 13.2, 19.4, 31.3, 32.1, 45.1, 114.2, 118.1, 123.8, 126.3, 128.8, 129.5, 132.9, 154.0, 154.1, 156.5, 169.6. Anal. calc. for C_46_H_52_N_10_: C, 74.16; H, 7.04; N, 18.80. Found: C, 74.11; H, 7.06; N, 18.83; HRMS (ESI): *m*/*z* calcd for C_46_H_52_N_10_+H^+^: 745.4455; found: 745.4454.
*3,6-bis(4-(4-butyl-5-(4-nitrophenyl)-4H-1,2,4-triazol-3-yl)phenyl)-1,2,4,5-tetrazine* (**13h**). The product was obtained as yellow powder (0.55 g, 51%); %); mp 189–190 °C. UV (CH_2_Cl_2_) λ_max_ (log ε) 294 (4.49) nm; IR (ATR) ν_max_ 3302, 3160, 2961, 2873, 2231, 1688, 1633, 1604, 1568, 1516, 1474, 1429, 1407, 1384, 1343, 1313, 1287, 1179, 1144, 1109, 1013, 975, 853, 761, 723, 693 cm^−1^; ^1^H-NMR (400 MHz, CDCl_3_): δ 0.68 (t, 6H, *J* = 8.0 Hz, CH_3_), 1.03 (sextet, 4H, *J* = 8.0 Hz, CH_2_), 1.39 (quintet, 4H, *J* = 8.0 Hz, CH_2_), 4.17 (t, 4H, *J* = 8.0 Hz, CH_2_), 7.86–7.91 (m, 8H, Ar) 7.92–7.94 (m, 4H, Ar), 8.42–8.44 (m, 4H, Ar); ^13^C-NMR (100 MHz, CDCl_3_): δ 13.2, 19.3, 31.9, 45.3, 114.4, 117.9, 124.3, 128.21, 129.5, 129.8, 132.9, 148.9, 154.2, 154.6, 168.2. Anal. calc. for C_38_H_34_N_12_O_4_: C, 63.15; H, 4.74; N, 23.26. Found: C, 63.12; H, 4.76; N, 23.25; HRMS (ESI): *m*/*z* calcd for C_38_H_34_N_12_O_4_+H^+^: 723.2904; found: 723.2902.

## 4. Conclusions

An efficient procedure for the synthesis of new extended *s*-tetrazine derivatives coupled via a 1,4-phenylene linker with a 4*H*-1,2,4-triazole ring has been presented. The methodology is useful for benzene ring moieties as well as for various types of substituents on the triazole nitrogen atom. Moreover, systems containing other five-membered rings can also be obtained in an analogous manner. The described approach resulted in a number of previously undescribed final products and intermediates. The synthesized *s*-tetrazine-4*H*-1,2,4-triazole derivatives exhibited fluorescent properties, which were dependent on the substituents bonded to the triazole rings. The correct selection of these substituents allowed the intentional tuning of absorption-emission properties such as emission wavelength, fluorescence intensity, or quantum yield.

## Figures and Tables

**Table 1:** The yield of the reaction for the preparation of imidoyl chlorides (**6a**–**d**) and 1,3,4-oxadiazoles (**7a**–**d**).

Entry	R^1^	Product 6	Yield [%]	Product 7	Yield [%]
1	H	6a	30	7a	40
2	OCH_3_	6b	22	7b	49
3	*t*-Bu	6c	26	7c	46
4	NO_2_	6d	n. r.	7d	57

**Table 2:** The yield of the reaction for the preparation of precursors containing a 4*H*-1,2,4-triazole ring (**9a**–**h**).

Entry	R^1^	R^2^	Product 10	Yield [%]	Product 9	Method A Yield [%]	Method B Yield [%]
1	H	Ph	**10a**	99	**9a**	78	81
2	OCH_3_	Ph	**10b**	82	**9b**	75	77
3	*t*-Bu	Ph	**10c**	82	**9c**	51	58
4	NO_2_	Ph	**10d**	83	**9d**	-	53
5	H	*n*-Bu	**10e**	86	**9e**	53	55
6	OCH_3_	*n*-Bu	**10f**	99	**9f**	76	75
7	*t*-Bu	*n*-Bu	**10g**	96	**9g**	87	86
8	NO_2_	*n*-Bu	**10h**	96	**9h**	-	51

**Table 3:** The yield of the reaction for the preparation of title derivatives **13a**–**h**.

Entry	R^1^	R^2^	Product 13	Solvent	Yield [%]
1	H	Ph	**13a**	toluene	43
2				ethanol	63
3	OCH_3_	Ph	**13b**	ethanol	95
4	*t*-Bu	Ph	**13c**	ethanol	73
5	NO_2_	Ph	**13d**	ethanol	55
6	H	*n*-Bu	**13e**	ethanol	64
7	OCH_3_	*n*-Bu	**13f**	ethanol	80
8	*t*-Bu	*n*-Bu	**13g**	ethanol	67
9	NO_2_	*n*-Bu	**13h**	ethanol	51

**Table 4:** Spectroscopic data for the studied s-tetrazine derivatives. λ_abs_—wavelength of absorption maximum directly preceding λ_em_. λ_ex_ and λ_em_—excitation and emission wavelength at global fluorescence maximum. Stokes shift was calculated as λ_em_ − λ_abs_. The quantum yields Φ were determined according to the method described by Brouwer [31] by comparison with two standards: quinine sulphate (qn-SO_4_^2−^) [32] and *trans*,*trans*-1,4-diphenyl-1,3-butadiene (dpb) [33].

Entry	Compound	λ_abs_ (nm)	λ_ex_ (nm)	λ_em_ (nm)	Stokes Shift (nm)	Φ	
						**qn-SO_4_^2−^**	**dpb**
1	**13a**	282	293	374	92	0.69	0.67
2	**13b**	289	308	409	120	>0.98 *	>0.98 *
3	**13c**	264	297	387	123	0.33	0.33
4	**13d**	304	305	405	101	0.02	0.02
5	**13e**	271	288	380	109	0.59	0.58
6	**13f**	273	291	408	135	0.49	0.48
7	**13g**	267	289	391	124	0.51	0.50
8	**13h**	293	305	408	115	0.02	0.02

* exact values of Φ larger than 0.98 cannot be determined due to nonlinearity of standard/sample dependence in the Φ region 0.98–1.00 [31].

## Data Availability

Not applicable.

## Supplementary Materials

The following supporting information can be downloaded at. Copies of the ^1^H-NMR, ^13^C-NMR, UV-Vis and fluorescent spectra of the title compounds are available in the online Supplementary Materials.

## Sample Availability

Samples of the compounds **9a**–**h** and **13a**–**h** are available from the authors.
