# Supplementary material for: Novel Conjugated s-Tetrazine Derivatives Bearing a 4H-1,2,4-Triazole Scaffold: Synthesis and Luminescent Properties
Source: Molecules. 2022 Jan 11;27(2):459. doi: 10.3390/molecules27020459 (PMC8779062; doi:10.3390/molecules27020459)
Supplement: Supplementary file 1 [file molecules-27-00459-s001.zip › molecules-1524371-supplementary.pdf]

Supplementary Material

Novel conjugated *s*-tetrazine derivatives bearing a 4*H*-1,2,4-triazole scaffold: synthesis and luminescent properties

Anna Maj [1], Agnieszka Kudelko [1]* and Marcin Świątkowski [2]

[1] *Department of Chemical Organic Technology and Petrochemistry, The Silesian University of Technology, Krzywoustego 4, PL-44100 Gliwice, Poland;*
[2] *Institute of General and Ecological Chemistry, Lodz University of Technology, Zeromskiego 116, PL-90924 Lodz, Poland;*
* *Correspondence: agnieszka.kudelko@polsl.pl*

Table of contents

1. ^{1}H and ^{13}C NMR spectra 3

2. UV-Vis absorption spectra of compounds **13a-h** 22

3. 3D fluorescence spectra of compounds **13a-h** and scatter plot presenting emission data 23

1. ^{1}H and ^{13}C NMR spectra

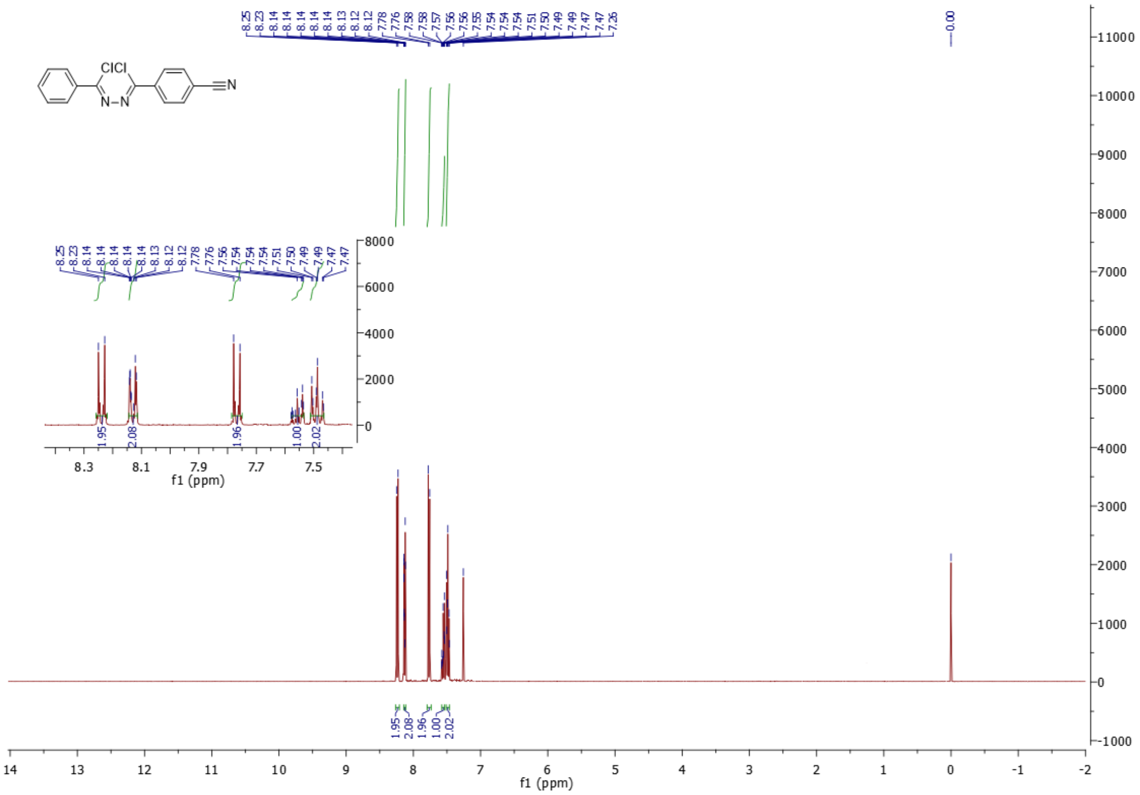

Figure S1. ^{1}H NMR spectra (400 MHz, $CDCl_3$) of *N*-chloro(phenyl)methylidene-4-cyanobenzene-1-carbohydrazonoyl chloride (**6a**)

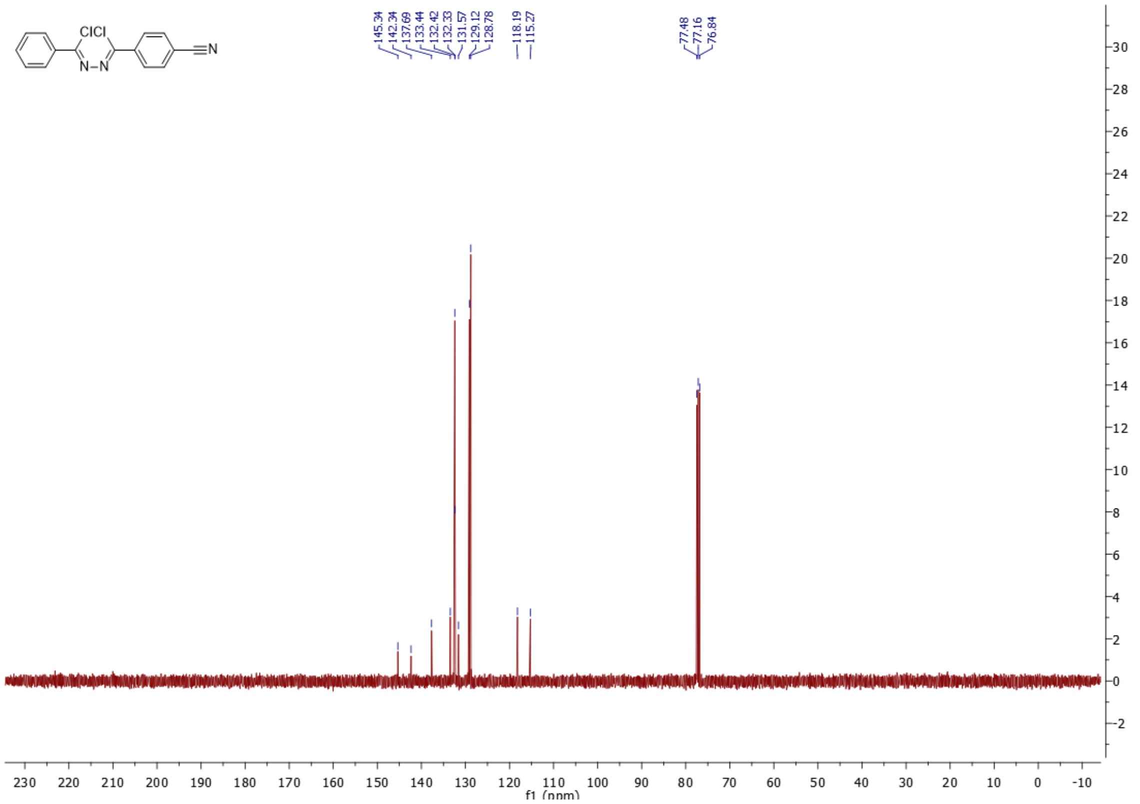

Figure S2. ^{13}C NMR spectra (100 MHz, $CDCl_3$) of *N*-chloro(phenyl)methylidene-4-cyanobenzene-1-carbohydrazonoyl chloride (**6a**)

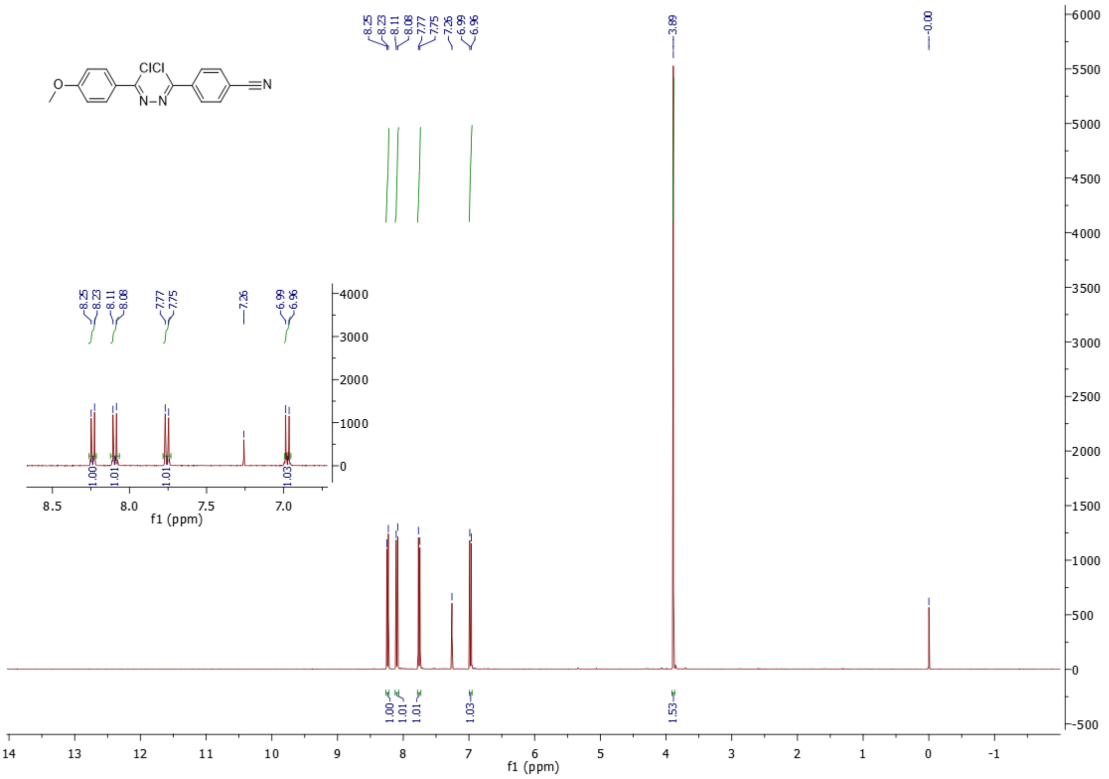

Figure S3. ^{1}H NMR spectra (400 MHz, $CDCl_3$) of *N*-chloro(4-methoxyphenyl)methylidene-4-cyanobenzene-1-carbohydrazonoyl chloride (**6b**)

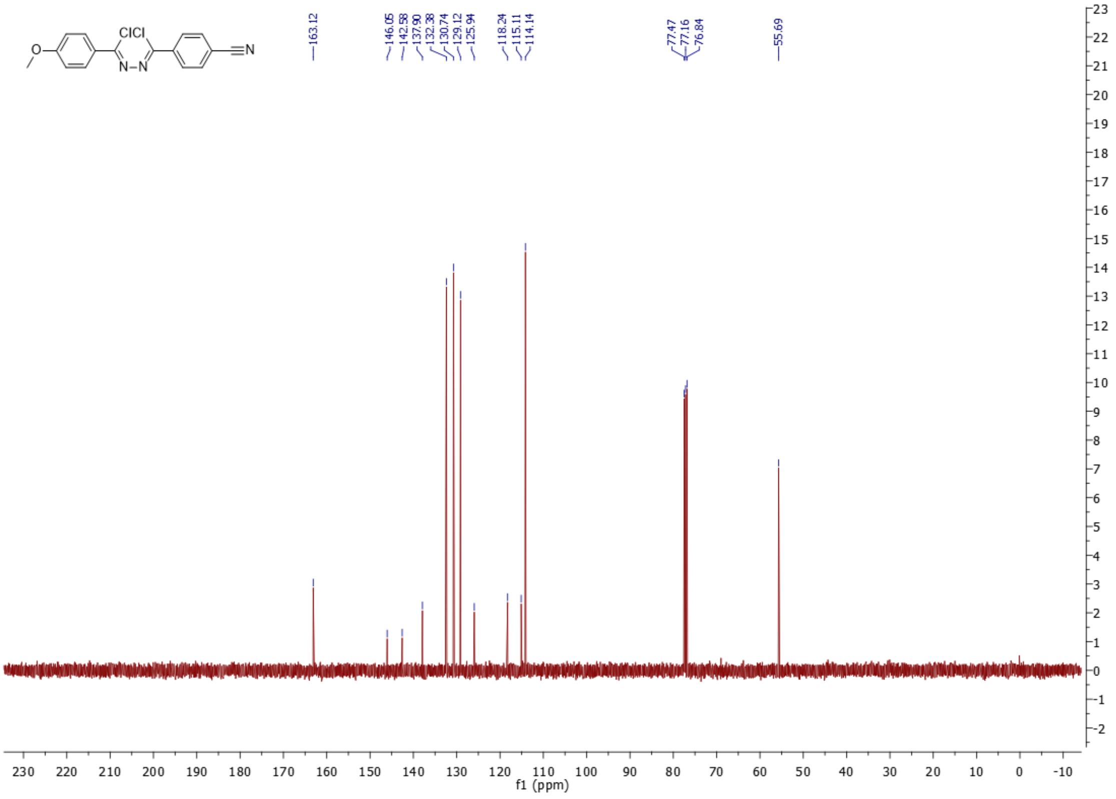

Figure S4. ^{13}C NMR spectra (100 MHz, $CDCl_3$) of *N*-chloro(4-methoxyphenyl)methylidene-4-cyanobenzene-1-carbohydrazonoyl chloride (**6b**)

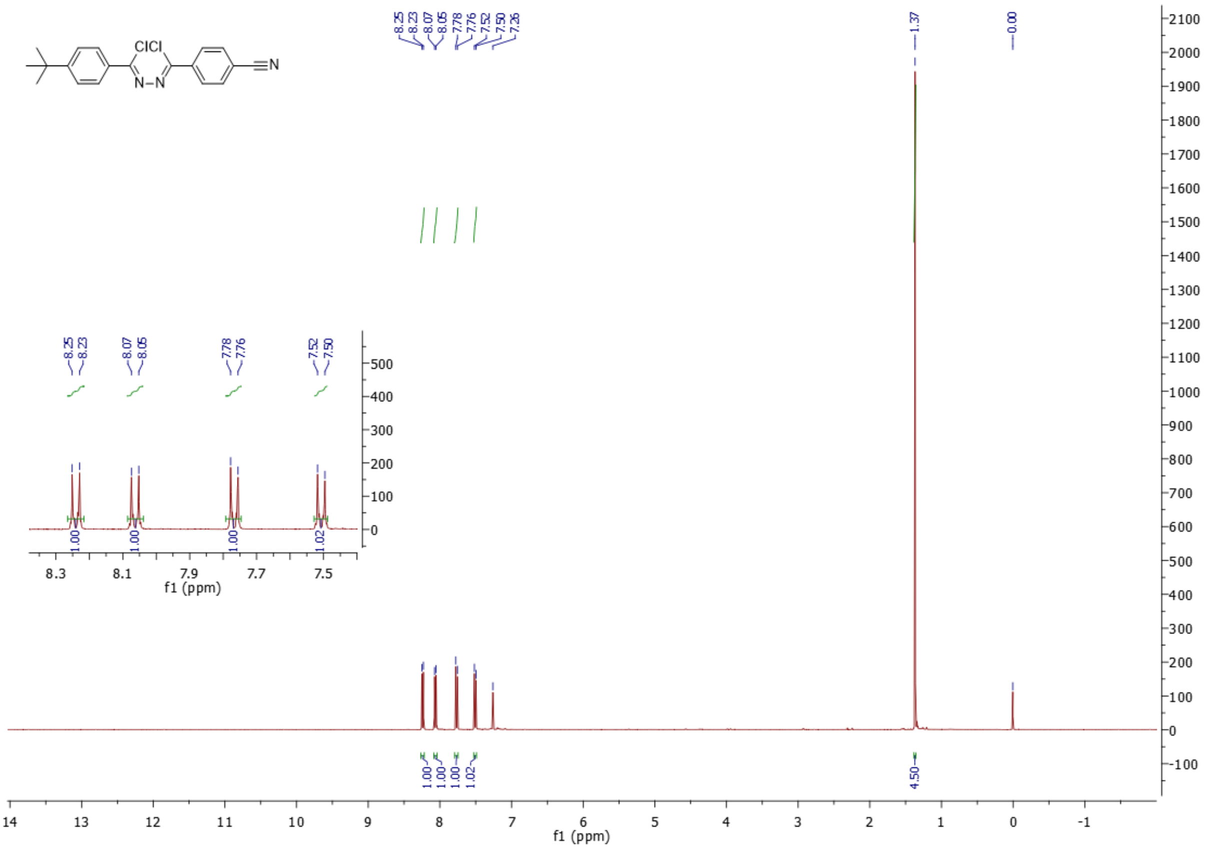

Figure S5. ^{1}H NMR spectra (400 MHz, $CDCl_3$) of 4-(*tert*-butyl)-*N*-(chloro(4-cyanophenyl)methylene)benzohydrazonoyl chloride (**6c**)

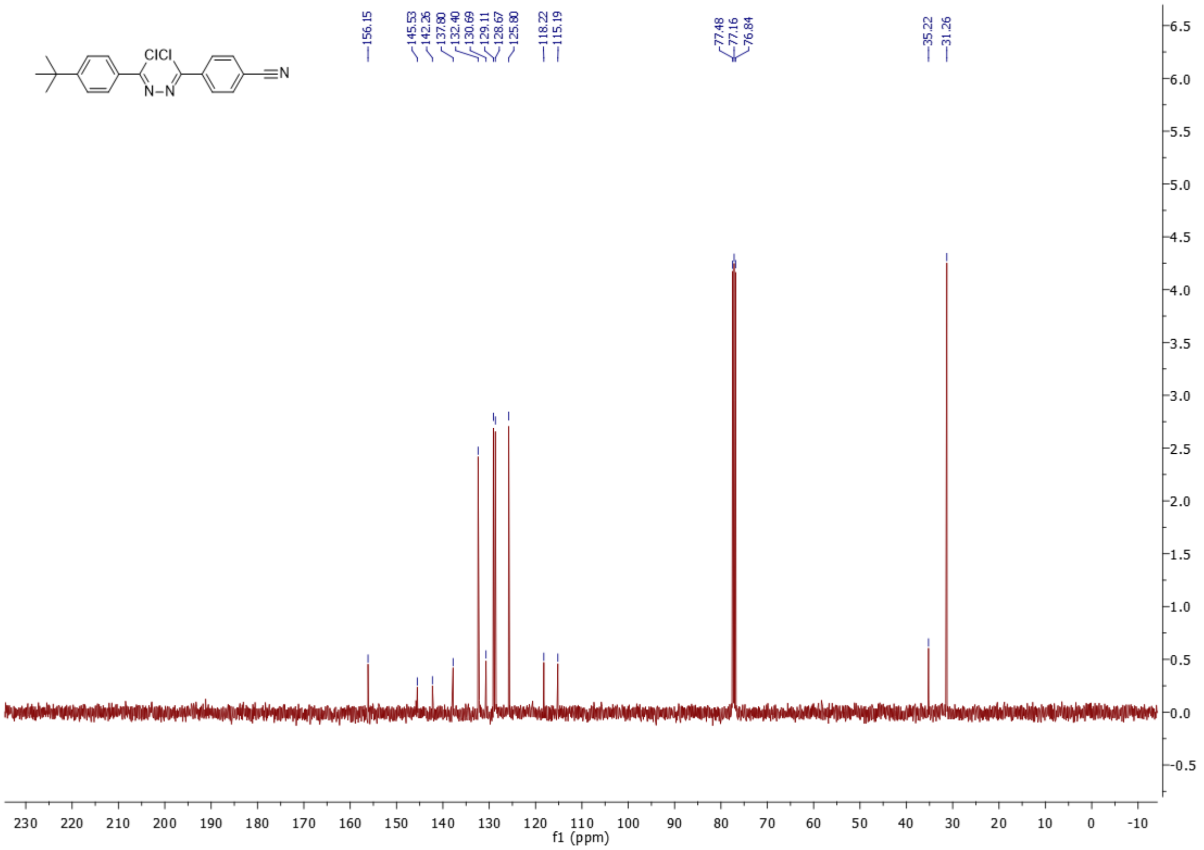

Figure S6. ^{13}C NMR spectra (100 MHz, $CDCl_3$) of 4-(*tert*-butyl)-*N*-(chloro(4-cyanophenyl)methylene)benzohydrazonoyl chloride (**6c**)

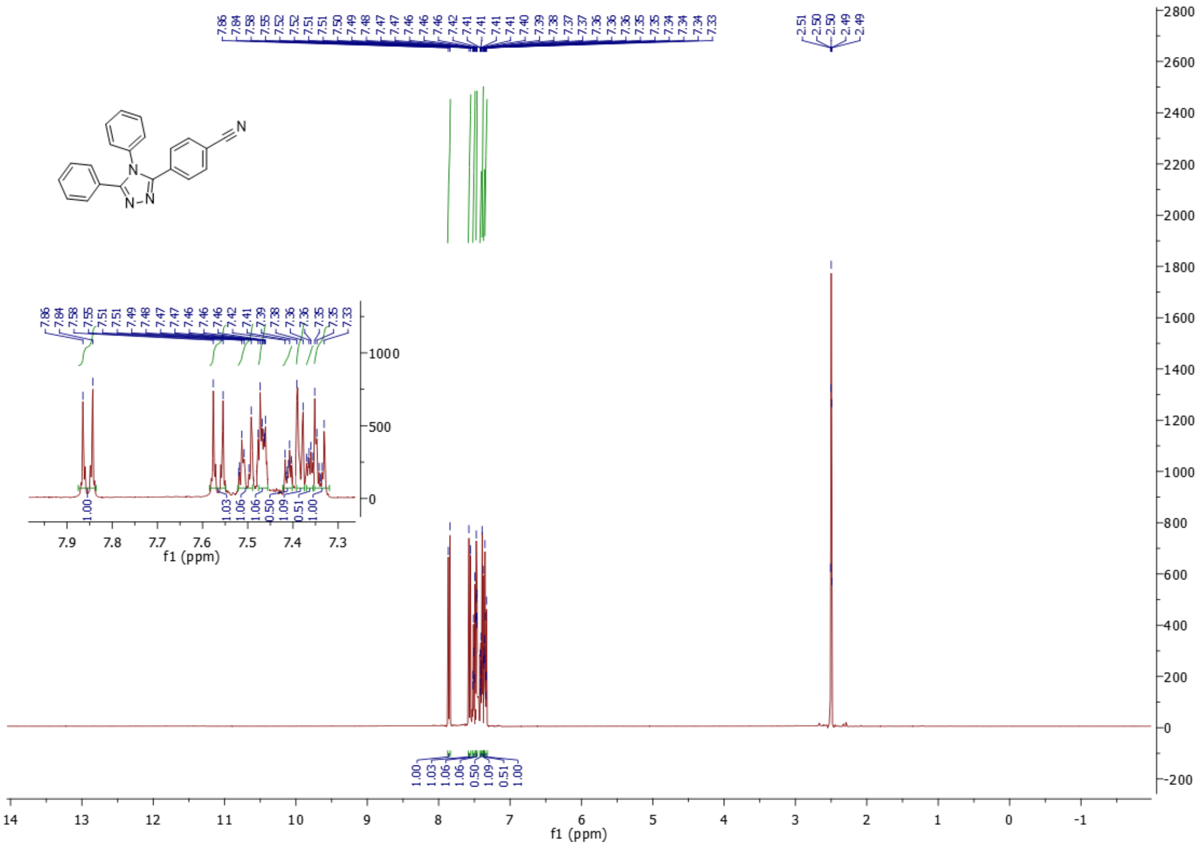

Figure S7. ^{1}H NMR spectra (400 MHz, DMSO-d_6) of 4-(4,5-diphenyl-4*H*-1,2,4-triazol-3-yl)benzonitrile (**9a**)

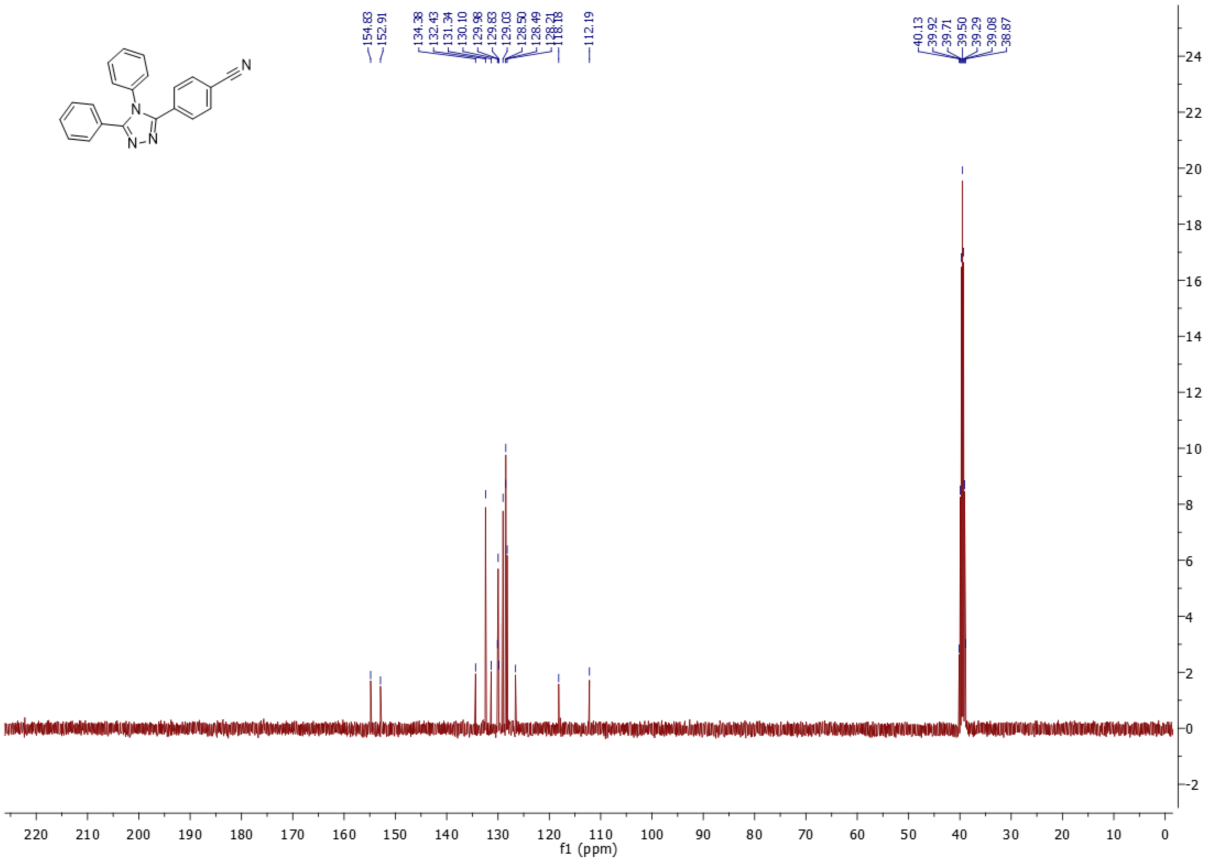

Figure S8. ^{13}C NMR spectra (100 MHz, DMSO-d_6) of 4-(4,5-diphenyl-4*H*-1,2,4-triazol-3-yl)benzonitrile (**8a**)

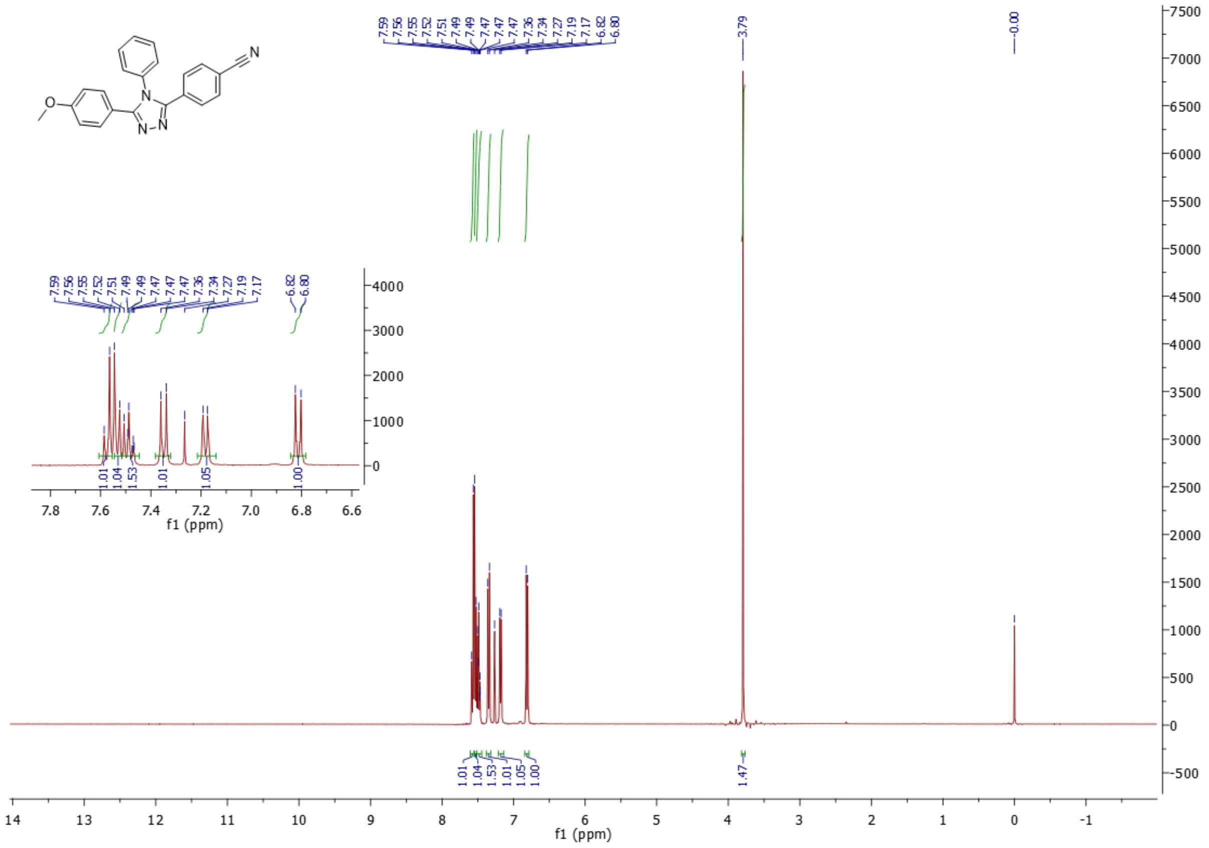

Figure S9. ^{1}H NMR spectra (400 MHz, $CDCl_3$) of 4-[5-(4-methoxyphenyl)-4-phenyl-4*H*-1,2,4-triazol-3-yl]benzonitrile (**9b**)

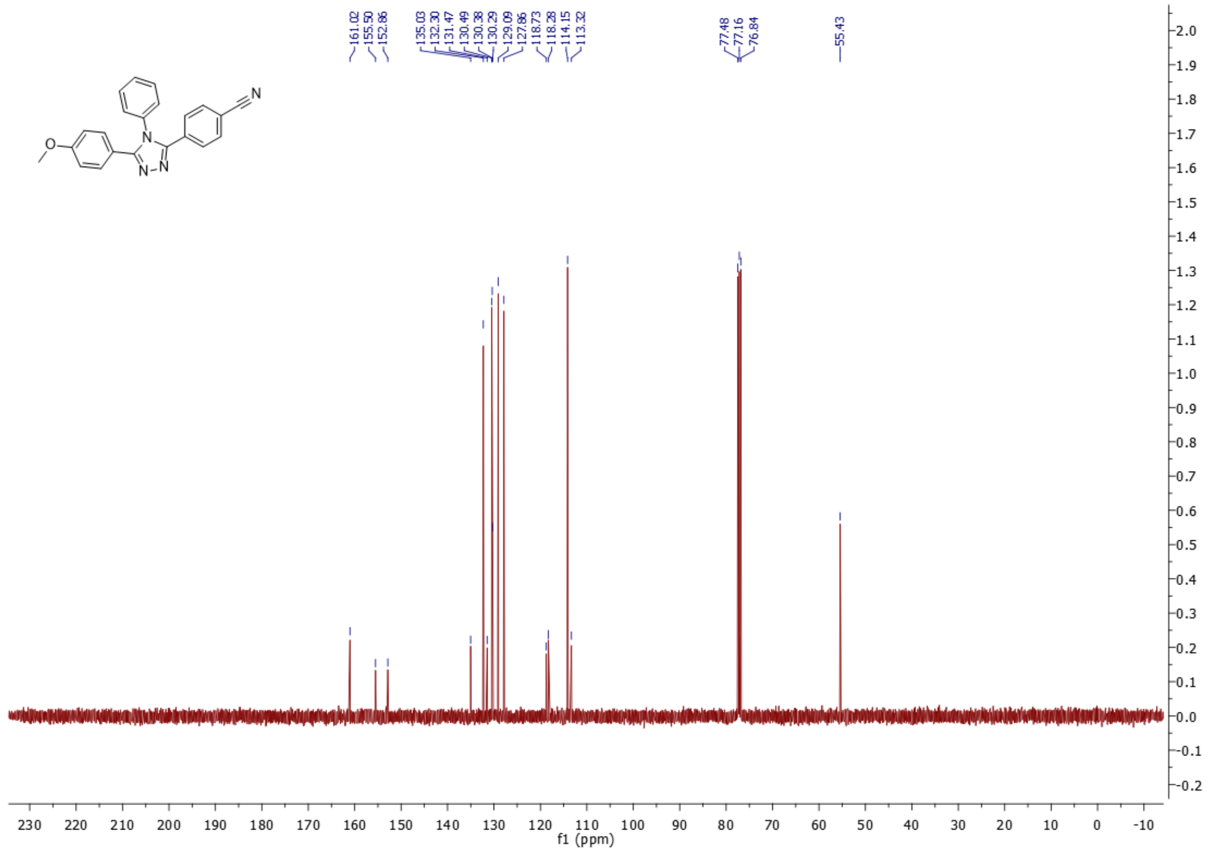

Figure S10. ^{13}C NMR spectra (100 MHz, $CDCl_3$) of 4-[5-(4-methoxyphenyl)-4-phenyl-4*H*-1,2,4-triazol-3-yl]benzonitrile (**9b**)

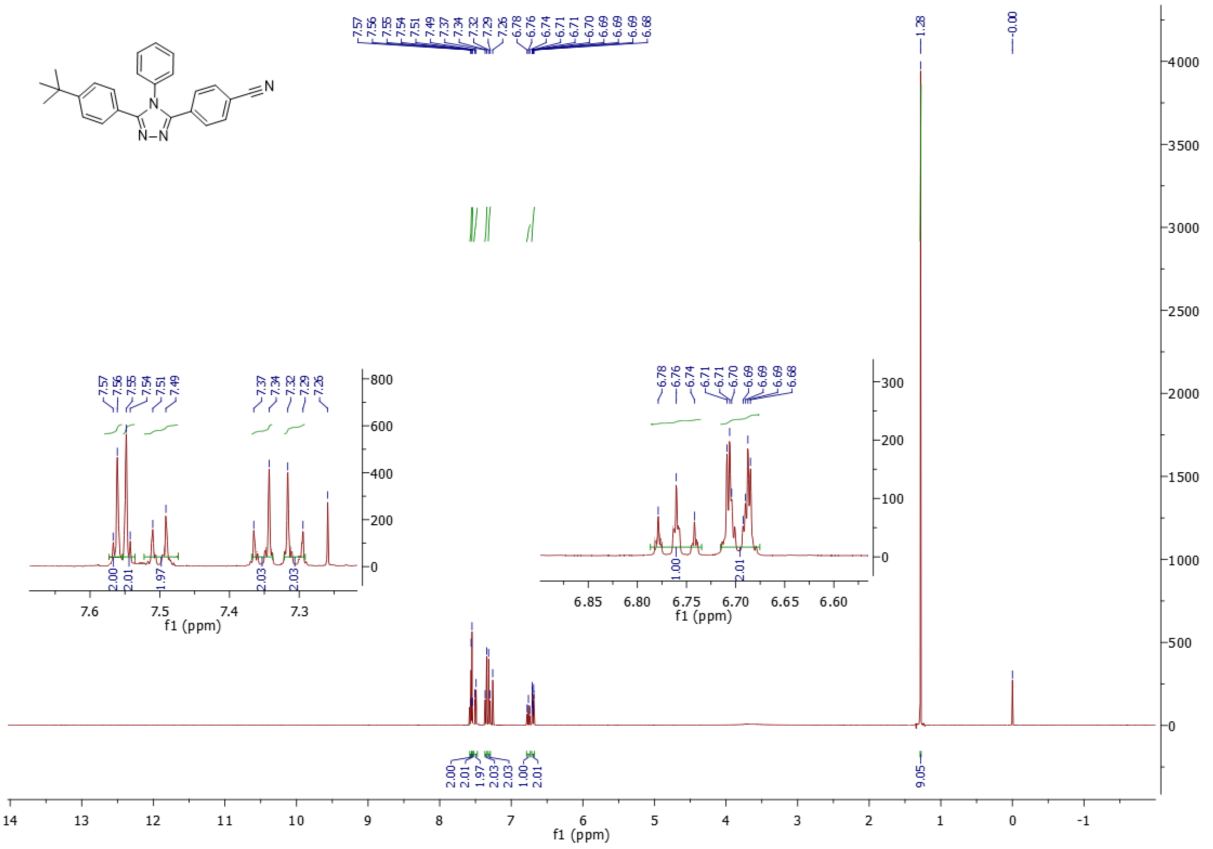

Figure S11. ^{1}H NMR spectra (400 MHz, $CDCl_3$) of 4-[5-(4-*tert*-butylphenyl)-4-phenyl-4*H*-1,2,4-triazol-3-yl]benzonitrile (**9c**)

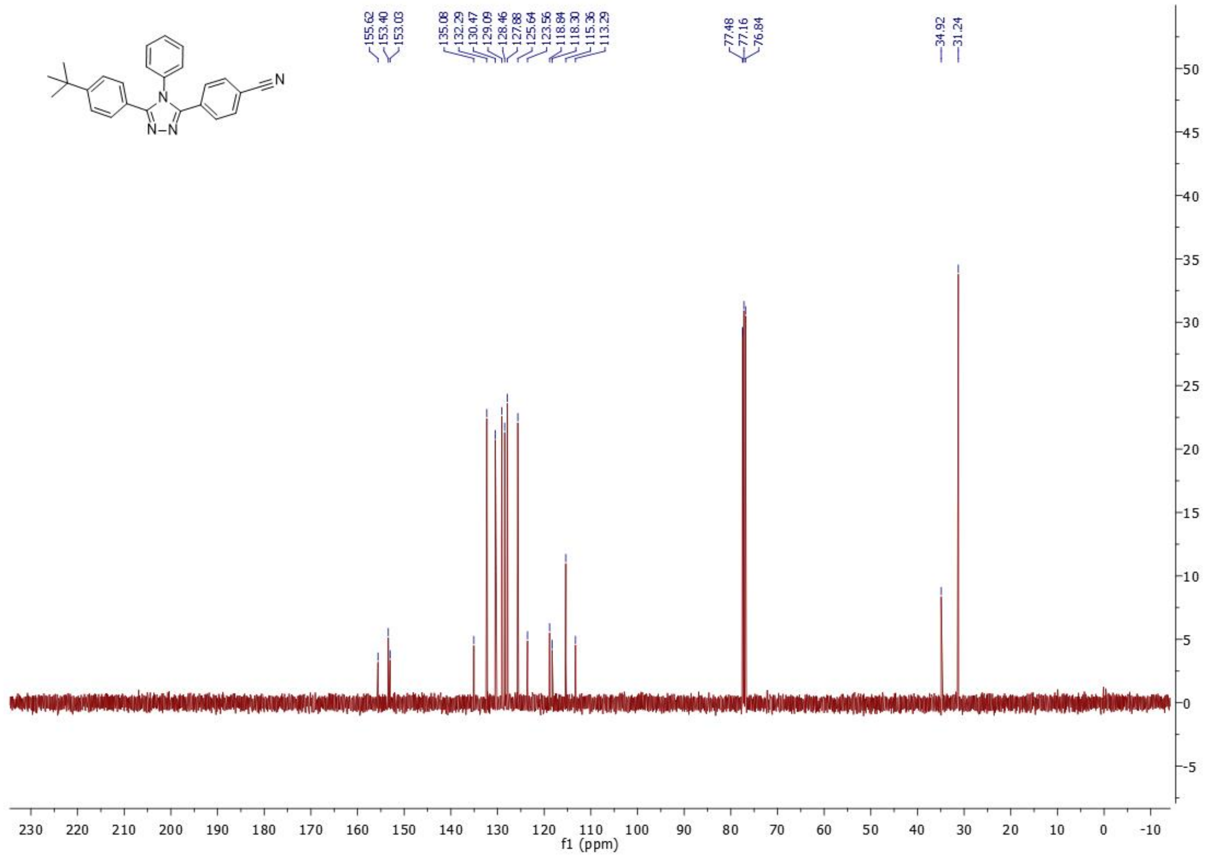

Figure S12. ^{13}C NMR spectra (100 MHz, $CDCl_3$) of 4-[5-(4-*tert*-butylphenyl)-4-phenyl-4*H*-1,2,4-triazol-3-yl]benzonitrile (**9c**)

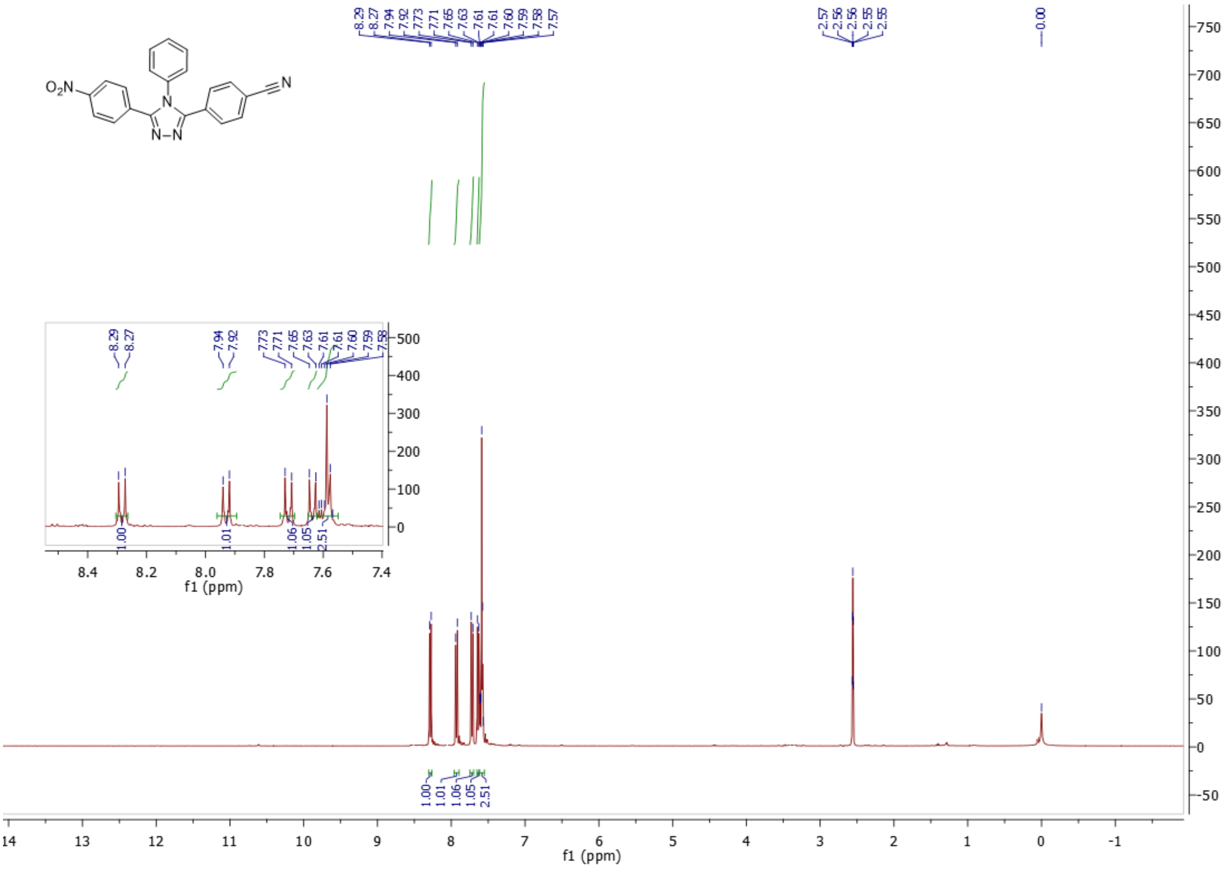

Figure S13. ^{1}H NMR spectra (400 MHz, DMSO-d$_6$) of 4-[5-(4-nitrophenyl)-4-phenyl-4*H*-1,2,4-triazol-3-yl]benzonitrile (**9d**)

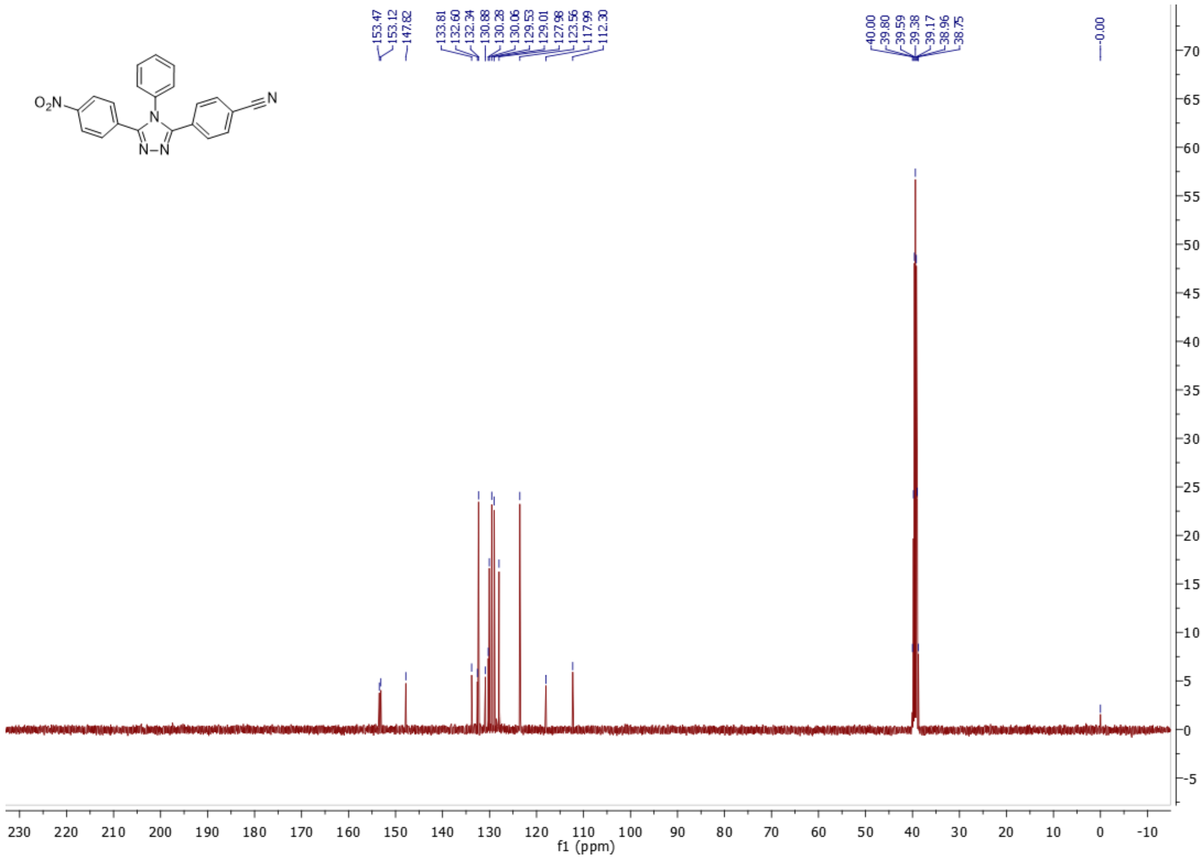

Figure S14. ^{13}C NMR spectra (100 MHz, DMSO-d$_6$) of 4-[5-(4-nitrophenyl)-4-phenyl-4*H*-1,2,4-triazol-3-yl]benzonitrile (**9d**)

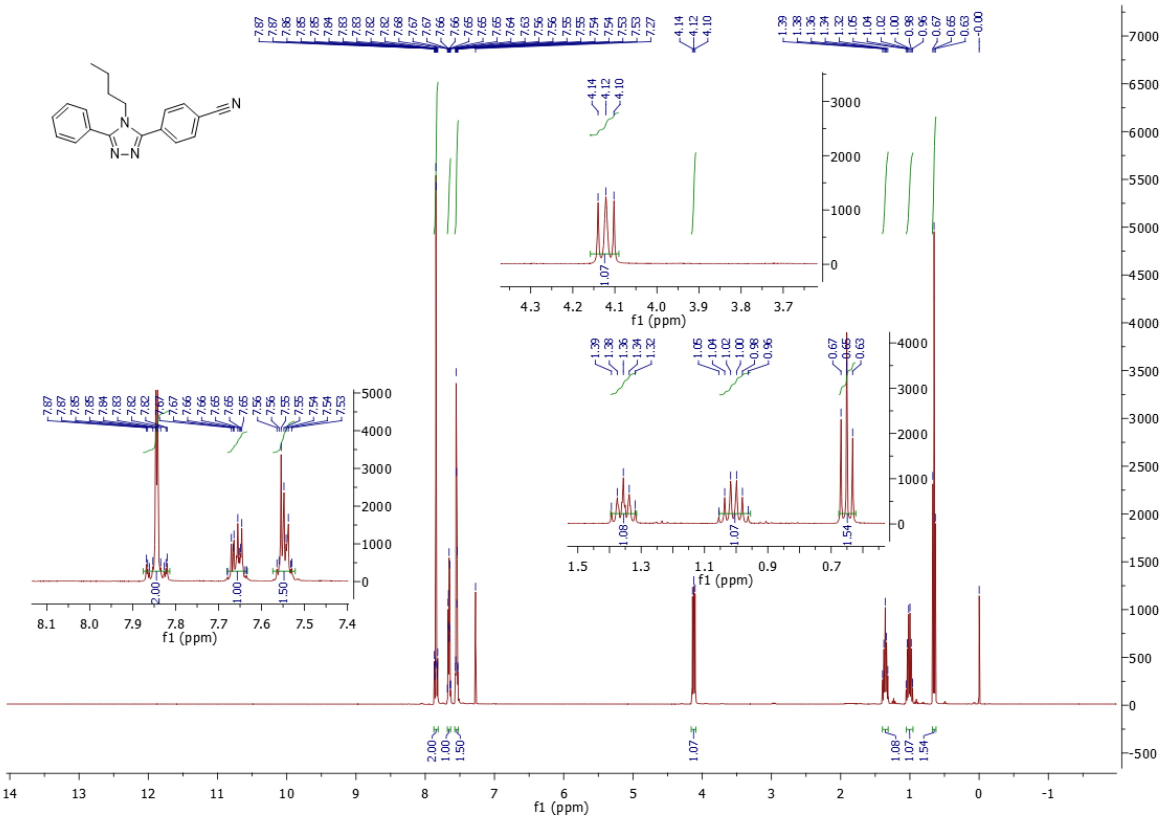

Figure S15. ^{1}H NMR spectra (400 MHz, $CDCl_3$) of 4-(4-butyl-5-phenyl-4*H*-1,2,4-triazol-3-yl)benzonitrile (**9e**)

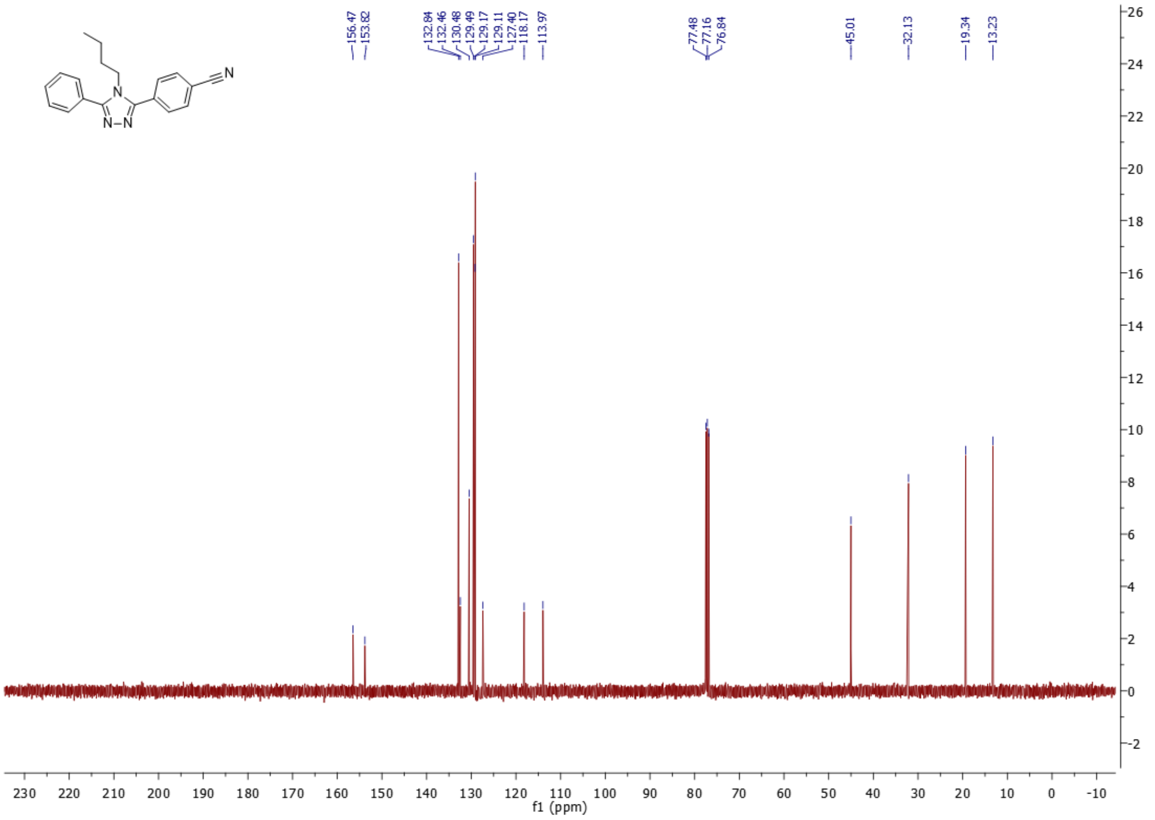

Figure S16. ^{13}C NMR spectra (100 MHz, $CDCl_3$) of 4-(4-butyl-5-phenyl-4*H*-1,2,4-triazol-3-yl)benzonitrile (**9e**)

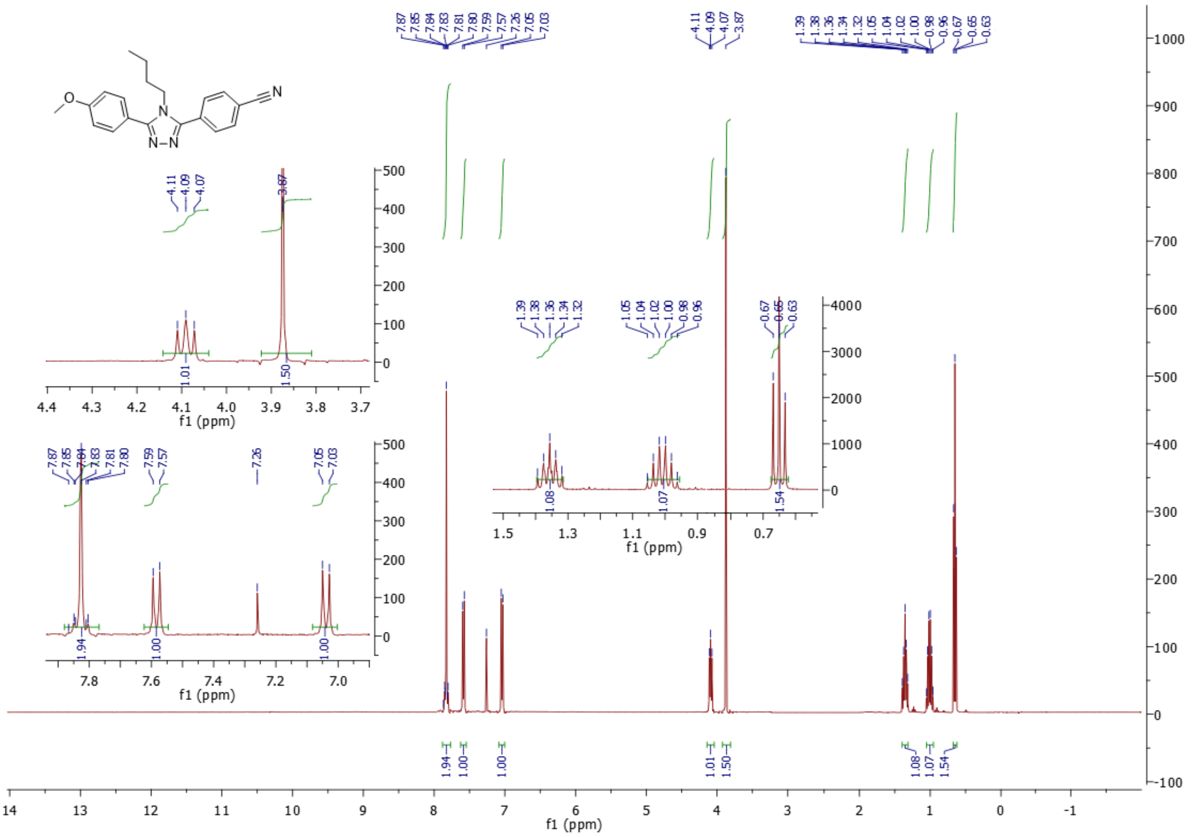

Figure S17. ^{1}H NMR spectra (400 MHz, $CDCl_3$) of 4-[4-butyl-5-(4-methoxyphenyl)-4*H*-1,2,4-triazol-3-yl]benzonitrile (**9f**)

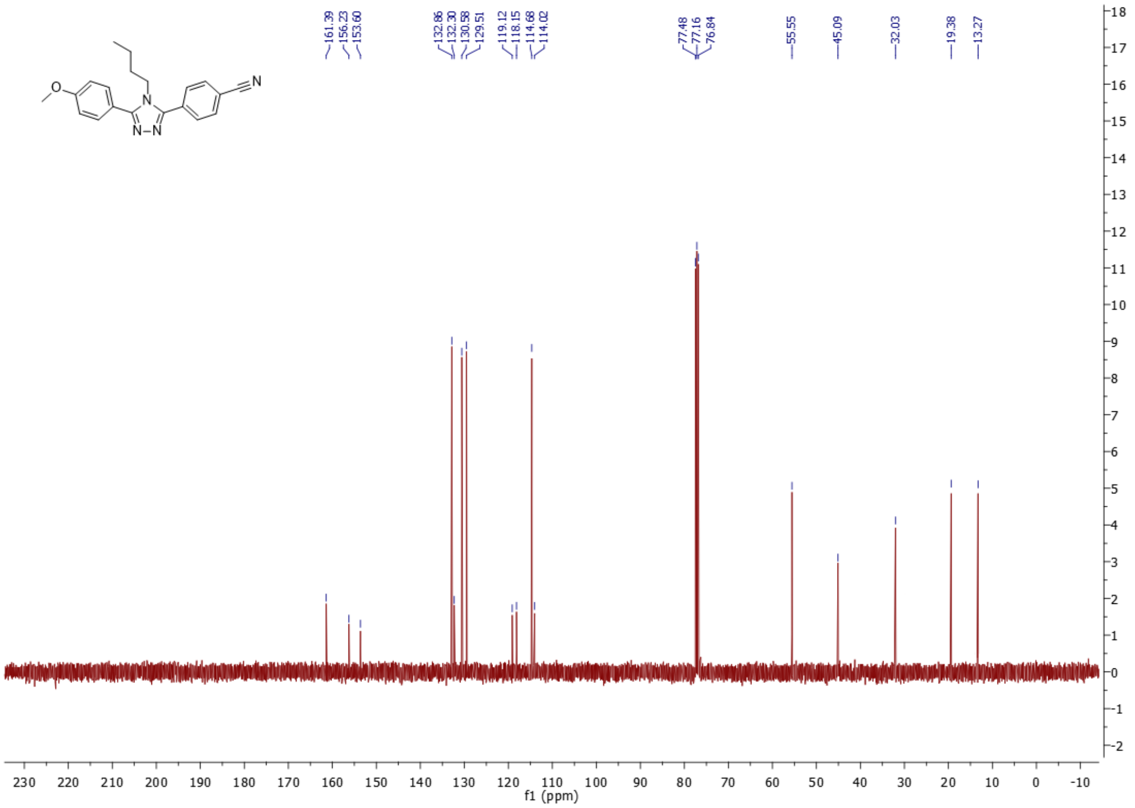

Figure S18. ^{13}C NMR spectra (100 MHz, $CDCl_3$) of 4-[4-butyl-5-(4-methoxyphenyl)-4*H*-1,2,4-triazol-3-yl]benzonitrile (**9f**)

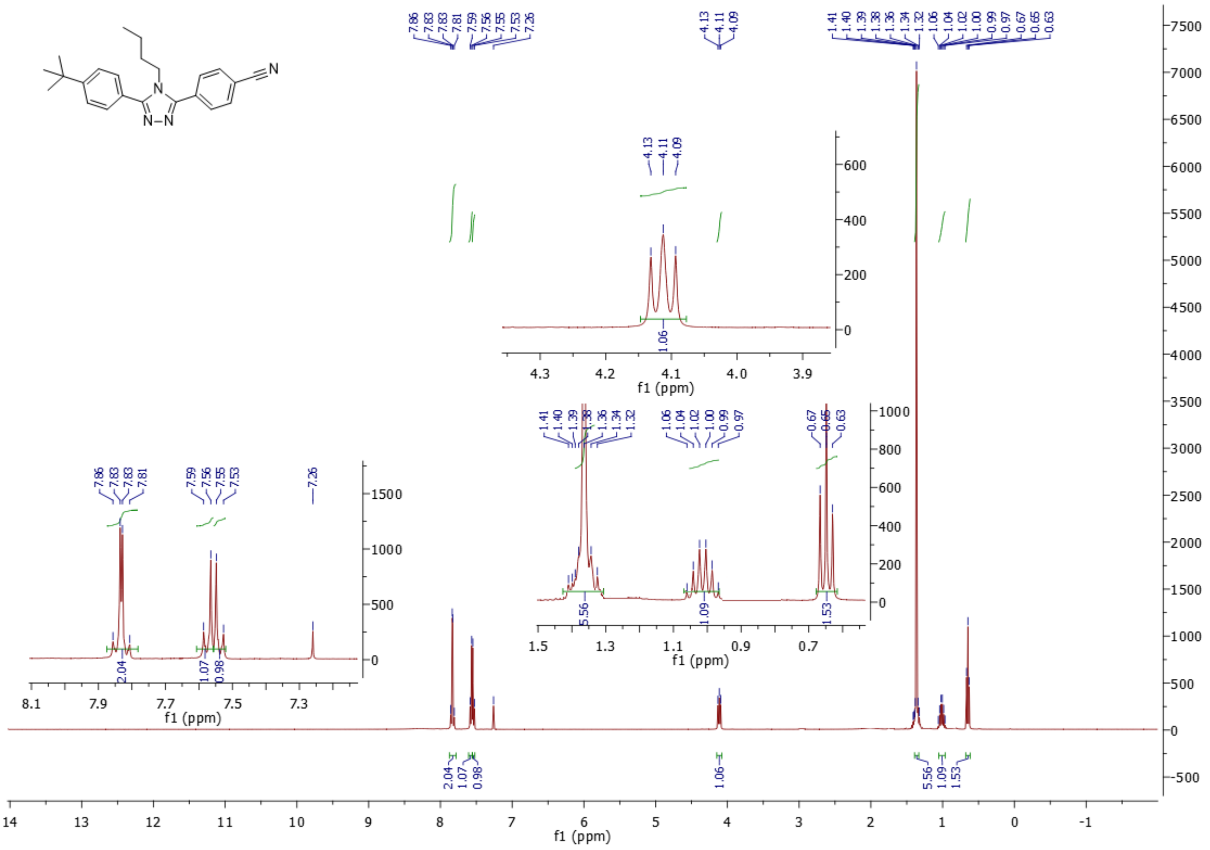

Figure S19. ^{1}H NMR spectra (400 MHz, $CDCl_3$) of 4-[4-butyl-5-(4-*tert*-butylphenyl)-4*H*-1,2,4-triazol-3-yl]benzonitrile (**9g**)

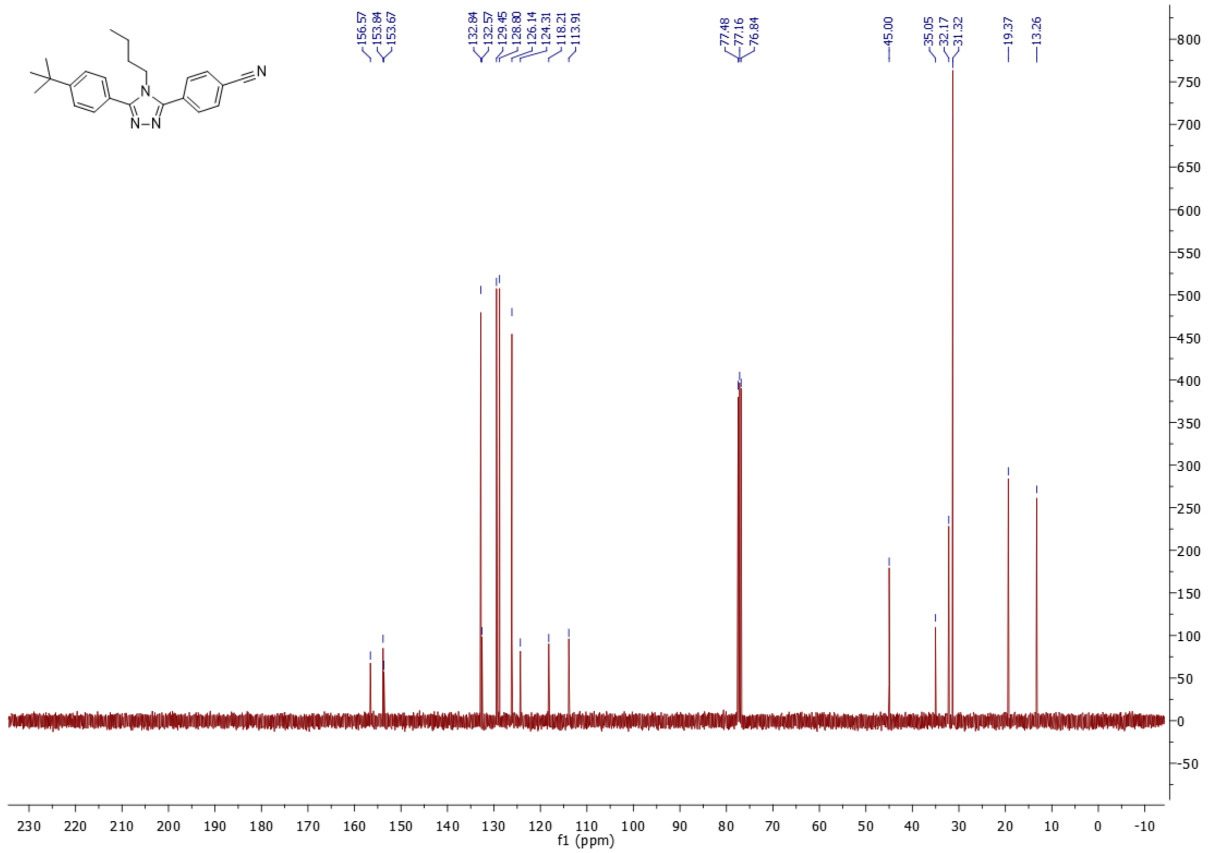

Figure S20. ^{13}C NMR spectra (100 MHz, $CDCl_3$) of 4-[4-butyl-5-(4-*tert*-butylphenyl)-4*H*-1,2,4-triazol-3-yl]benzonitrile (**9g**)

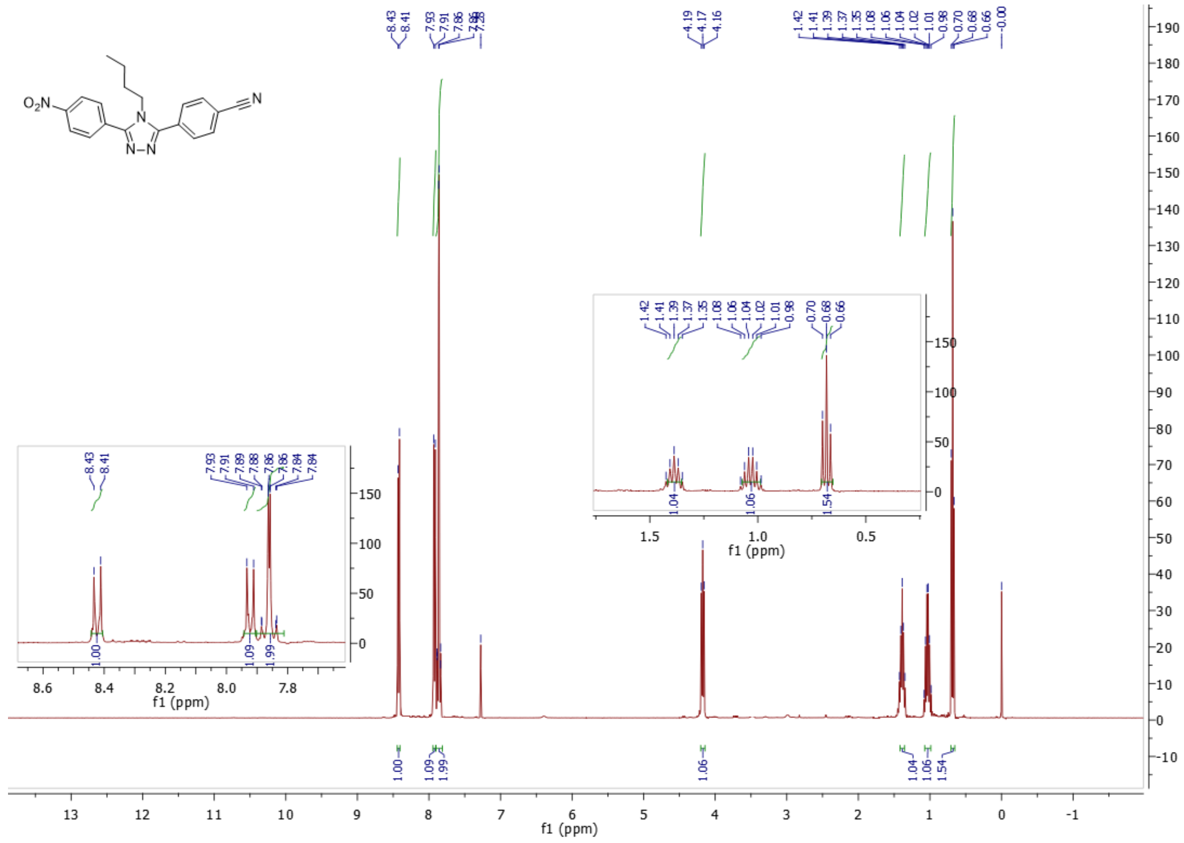

Figure S21. ^{1}H NMR spectra (400 MHz, $CDCl_3$) of 4-[4-butyl-5-(4-nitrophenyl)-4*H*-1,2,4-triazol-3-yl]benzonitrile (**9h**)

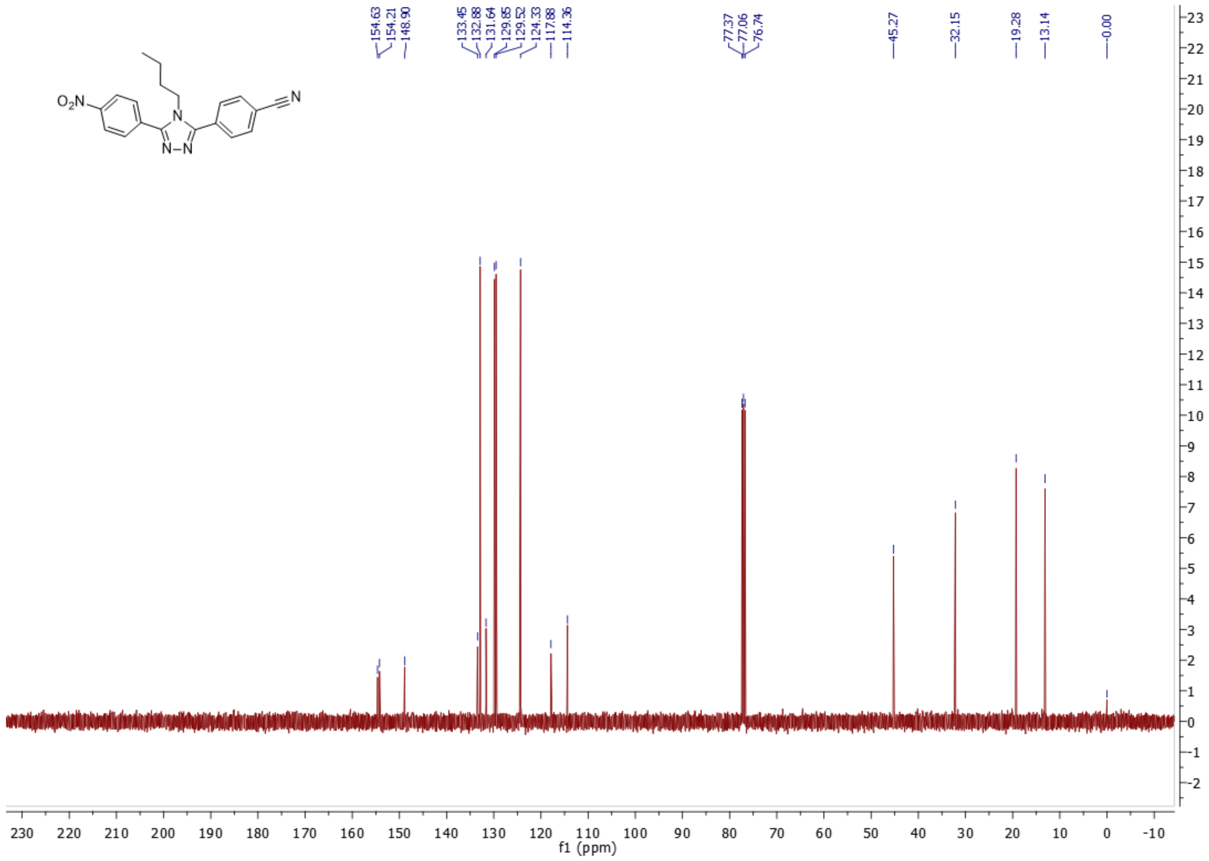

Figure S22. ^{13}C NMR spectra (100 MHz, $CDCl_3$) of 4-[4-butyl-5-(4-nitrophenyl)-4*H*-1,2,4-triazol-3-yl]benzonitrile (**9h**)

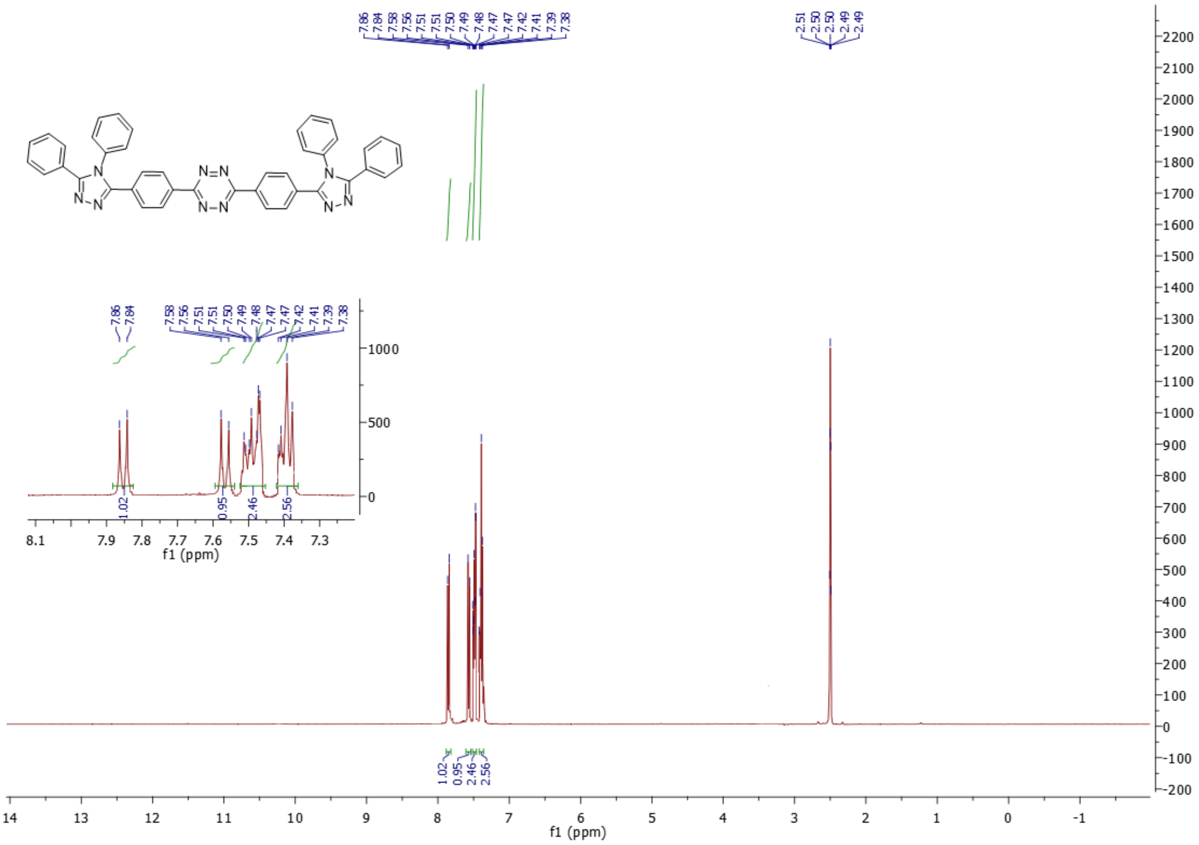

Figure S23. ^{1}H NMR spectra (400 MHz, DMSO-d_6) of 3,6-bis(4-(4,5-diphenyl-4*H*-1,2,4-triazol-3-yl)phenyl)-1,2,4,5-tetrazine (**13a**)

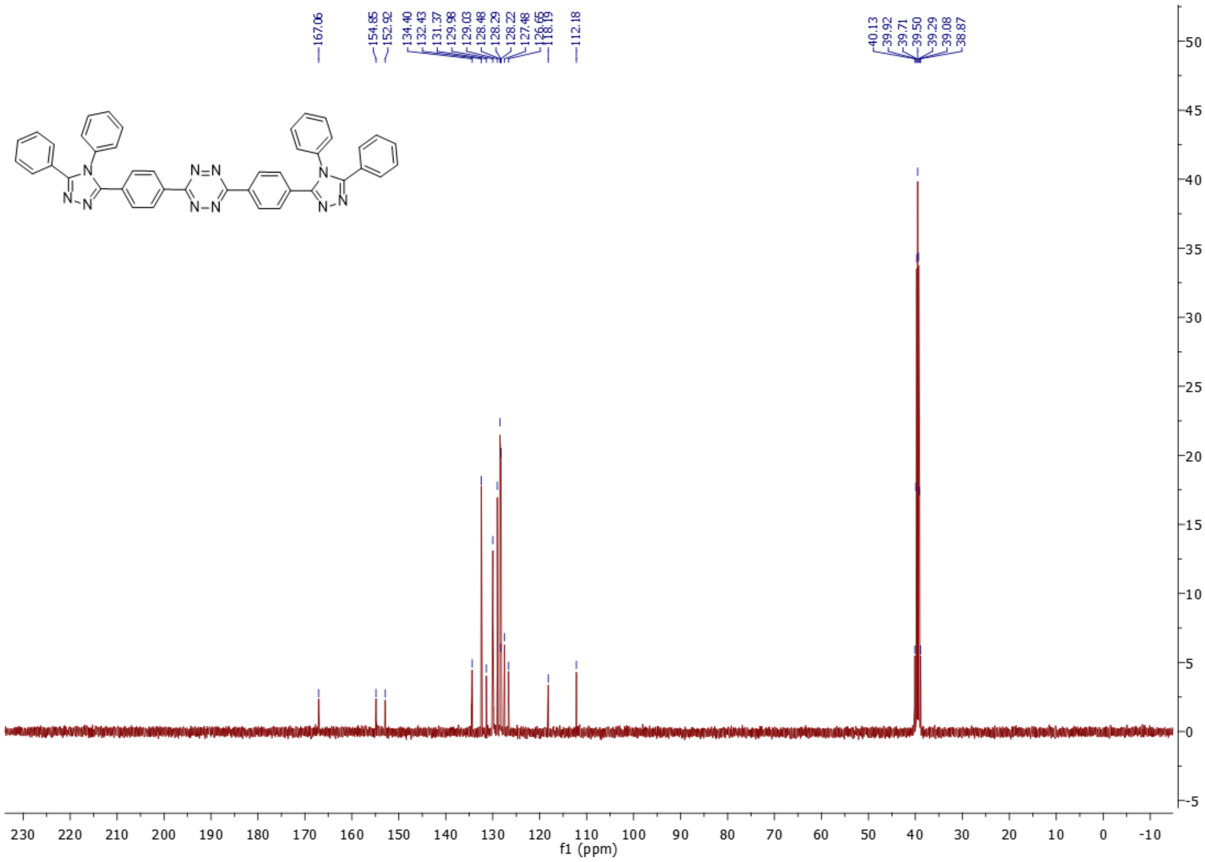

Figure S24. ^{13}C NMR spectra (100 MHz, DMSO-d_6) of 3,6-bis(4-(4,5-diphenyl-4*H*-1,2,4-triazol-3-yl)phenyl)-1,2,4,5-tetrazine (**13a**)

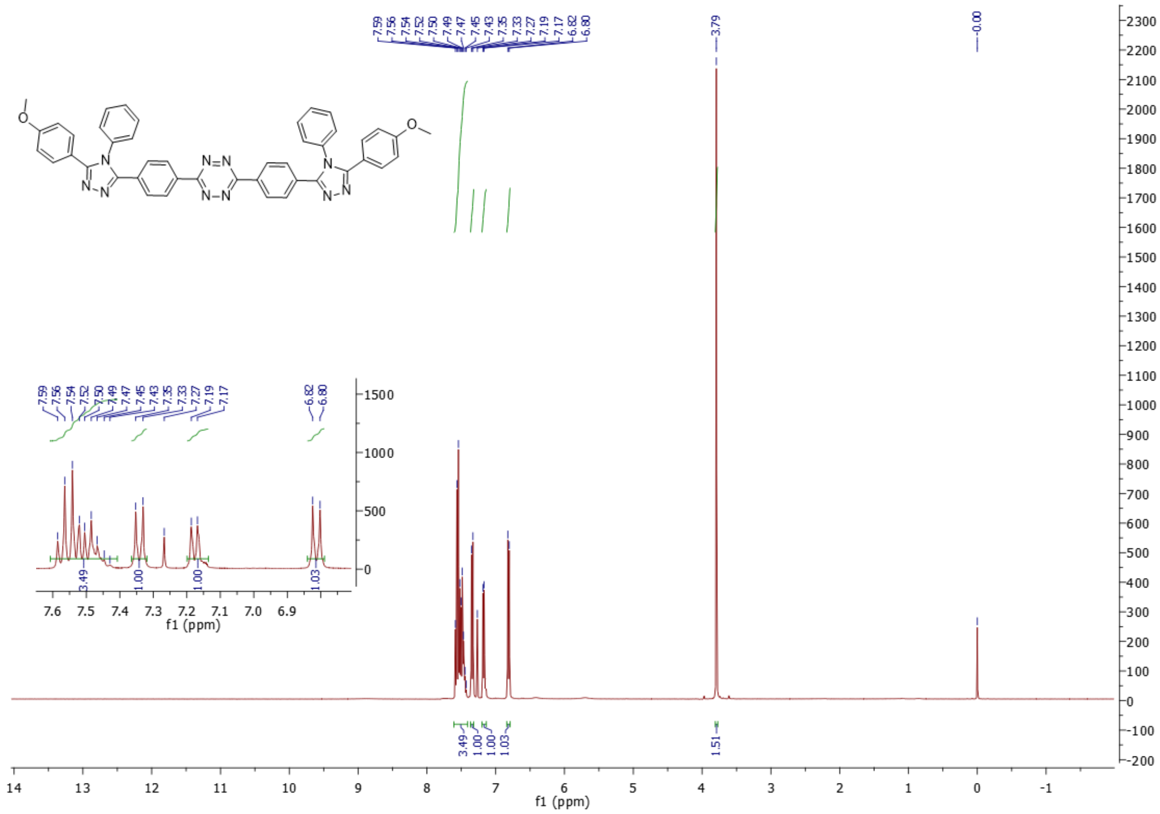

Figure S25. ^{1}H NMR spectra (400 MHz, $CDCl_3$) of 3,6-bis(4-(5-(4-methoxyphenyl)-4-phenyl-4*H*-1,2,4-triazol-3-yl)phenyl)-1,2,4,5-tetrazine (**13b**)

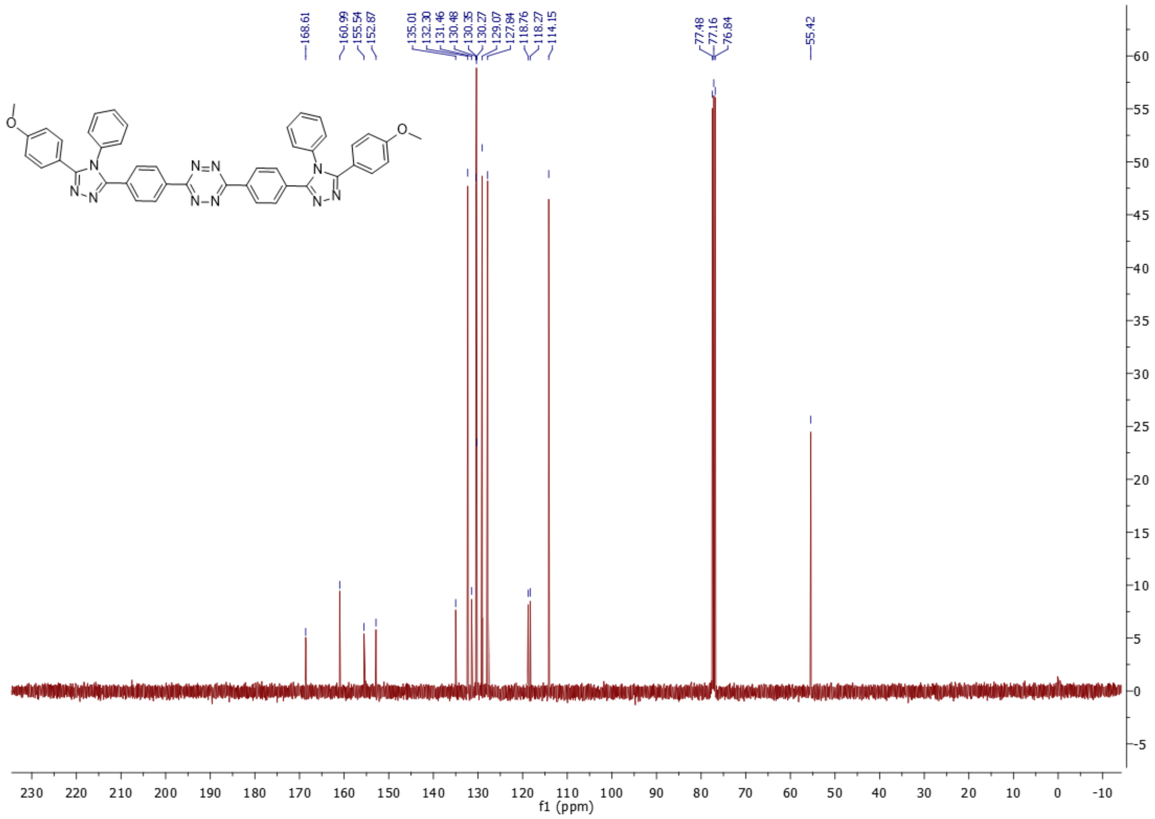

Figure S26. ^{13}C NMR spectra (100 MHz, $CDCl_3$) of 3,6-bis(4-(5-(4-methoxyphenyl)-4-phenyl-4*H*-1,2,4-triazol-3-yl)phenyl)-1,2,4,5-tetrazine (**13b**)

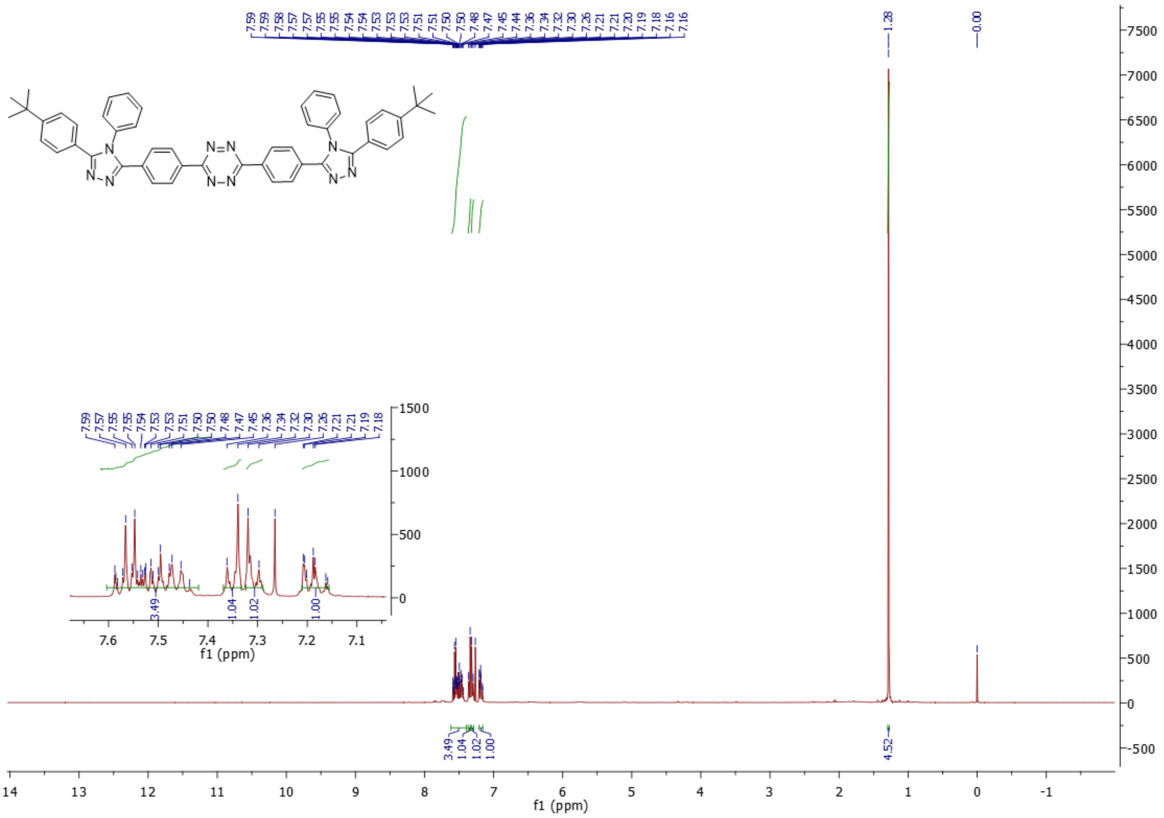

Figure S27. ^{1}H NMR spectra (400 MHz, $CDCl_3$) of 3,6-bis(4-(5-(4-(*tert*-butyl)phenyl)-4-phenyl-4*H*-1,2,4-triazol-3-yl)phenyl)-1,2,4,5-tetrazine (**13c**)

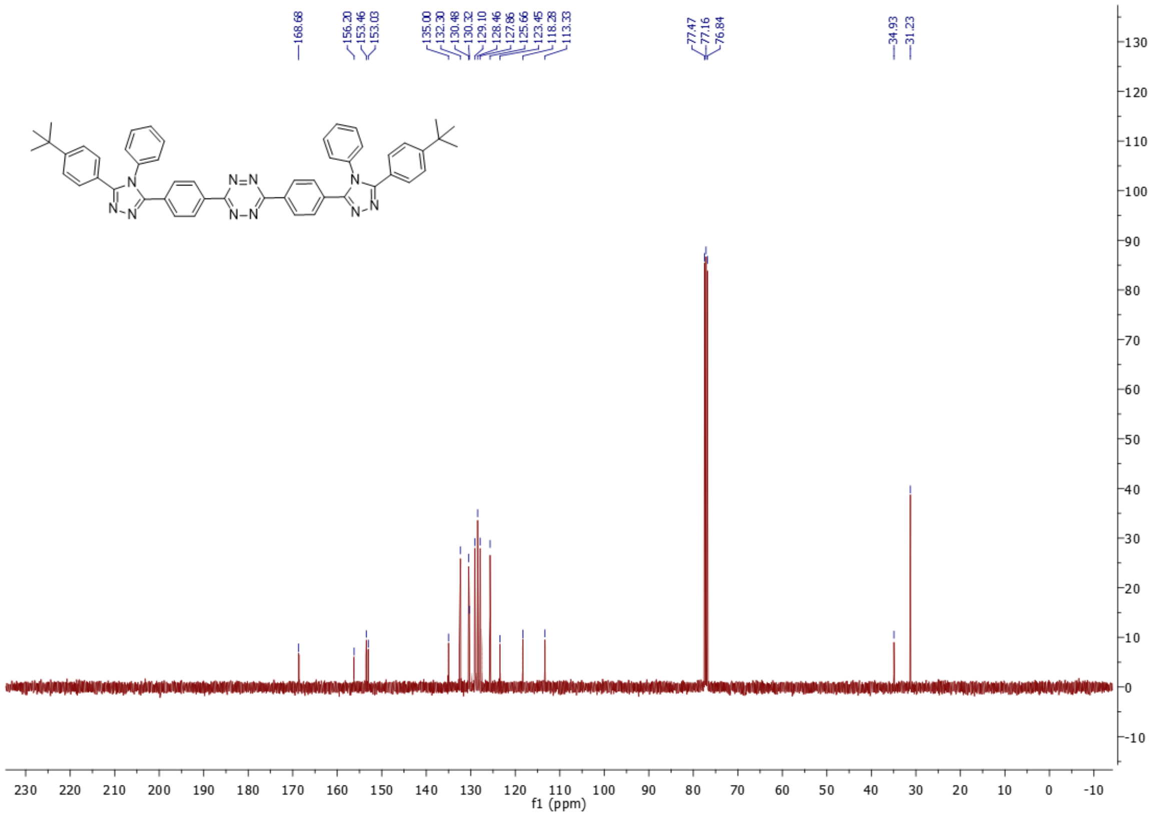

Figure S28. ^{13}C NMR spectra (100 MHz, $CDCl_3$) of 3,6-bis(4-(5-(4-(*tert*-butyl)phenyl)-4-phenyl-4*H*-1,2,4-triazol-3-yl)phenyl)-1,2,4,5-tetrazine (**13c**)

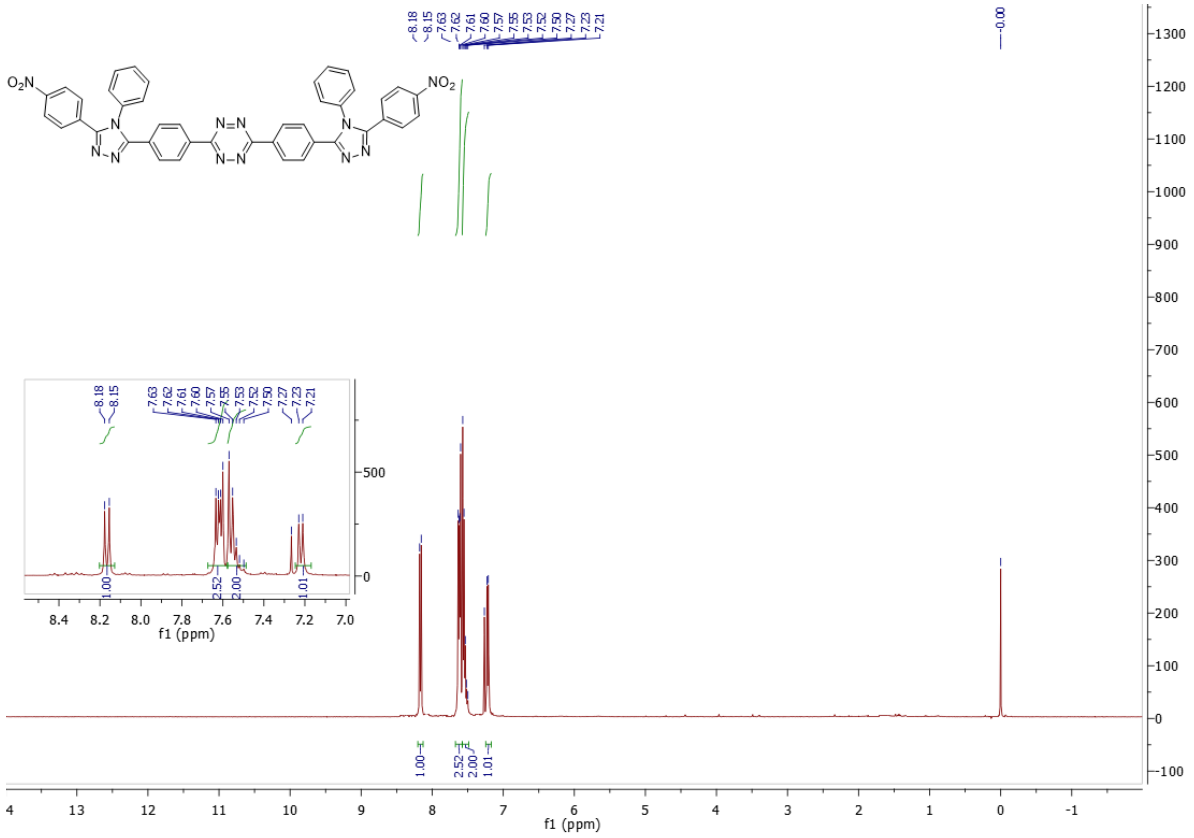

Figure S29. ^{1}H NMR spectra (400 MHz, $CDCl_3$) of 3,6-bis(4-(5-(4-nitrophenyl)-4-phenyl-4*H*-1,2,4-triazol-3-yl)phenyl)-1,2,4,5-tetrazine (**13d**)

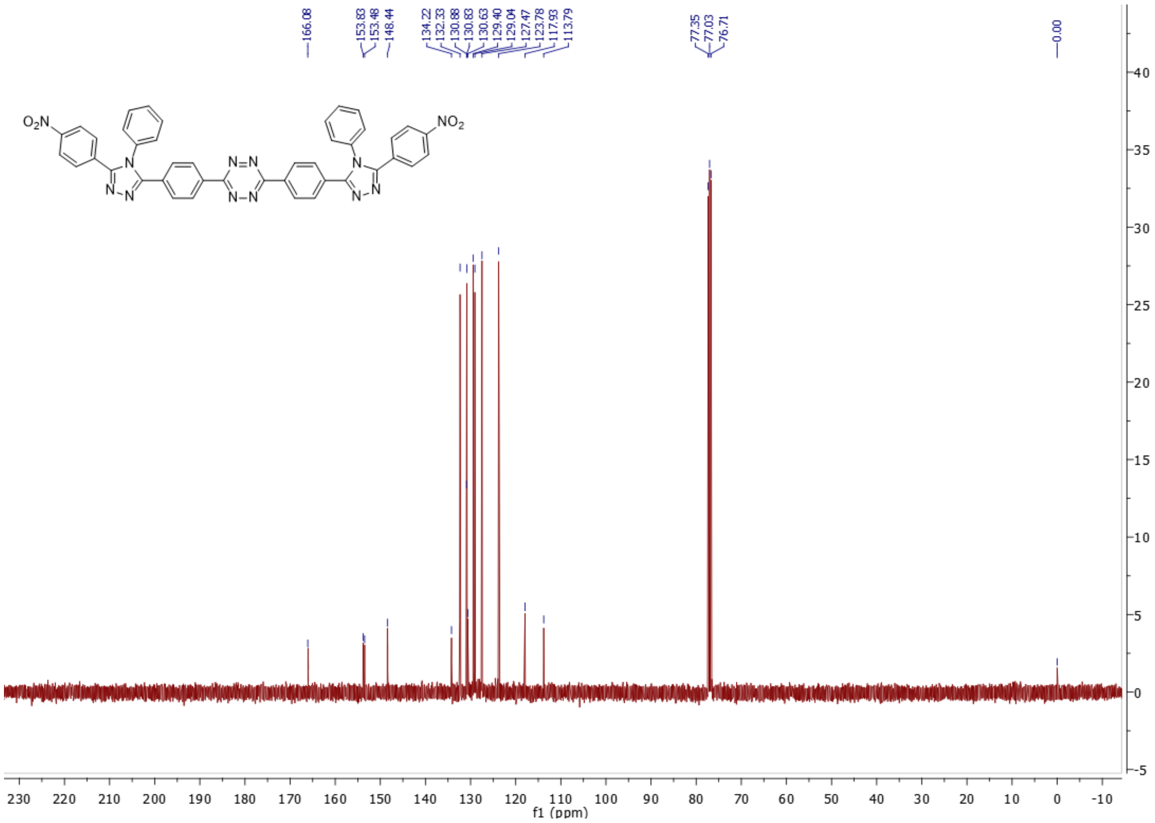

Figure S30. ^{13}C NMR spectra (100 MHz, $CDCl_3$) of 3,6-bis(4-(5-(4-nitrophenyl)-4-phenyl-4*H*-1,2,4-triazol-3-yl)phenyl)-1,2,4,5-tetrazine (**13d**)

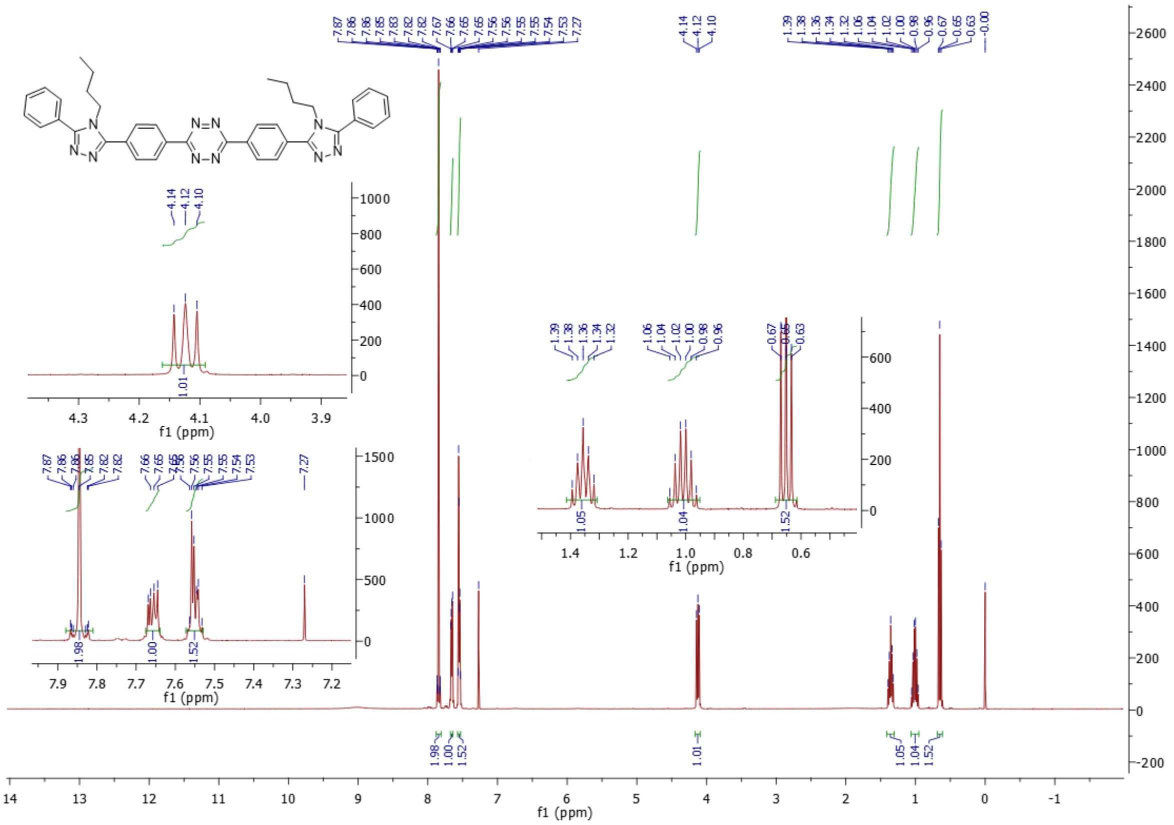

Figure S31. ^{1}H NMR spectra (400 MHz, $CDCl_3$) of 3,6-bis(4-(4-butyl-5-phenyl-4*H*-1,2,4-triazol-3-yl)phenyl)-1,2,4,5-tetrazine (**13e**)

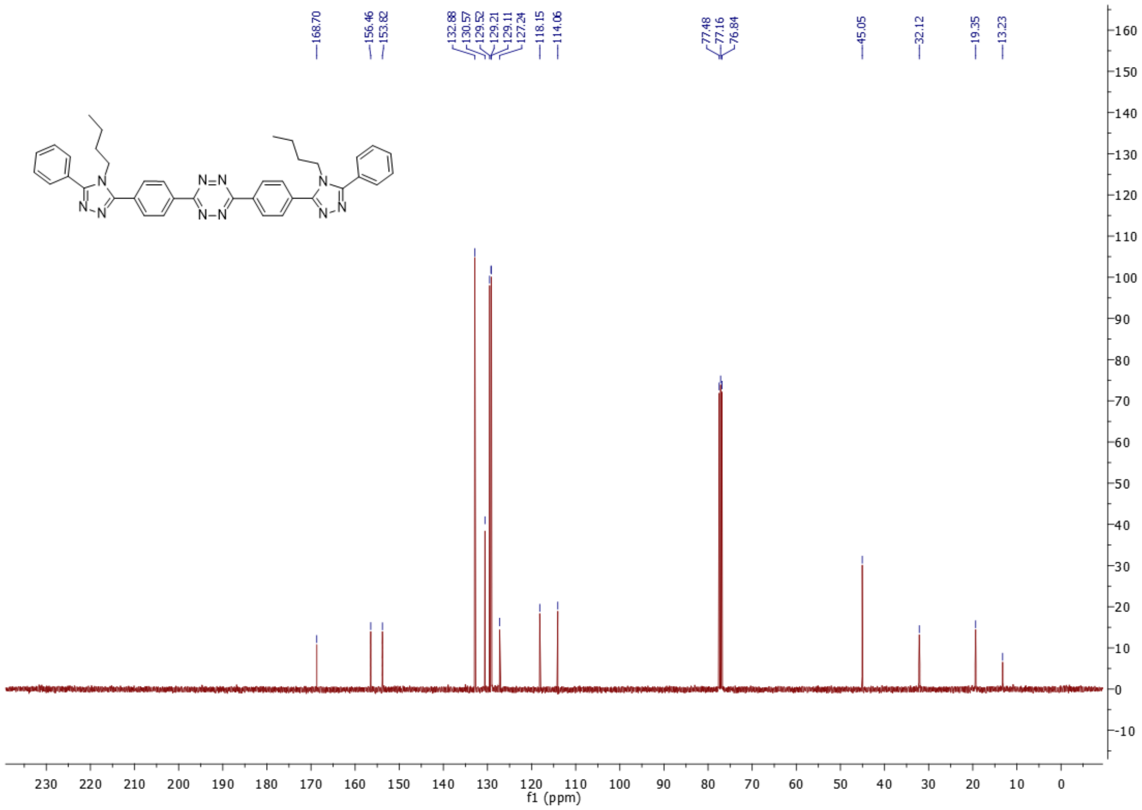

Figure S32. ^{13}C NMR spectra (100 MHz, $CDCl_3$) of 3,6-bis(4-(4-butyl-5-phenyl-4*H*-1,2,4-triazol-3-yl)phenyl)-1,2,4,5-tetrazine (**13e**)

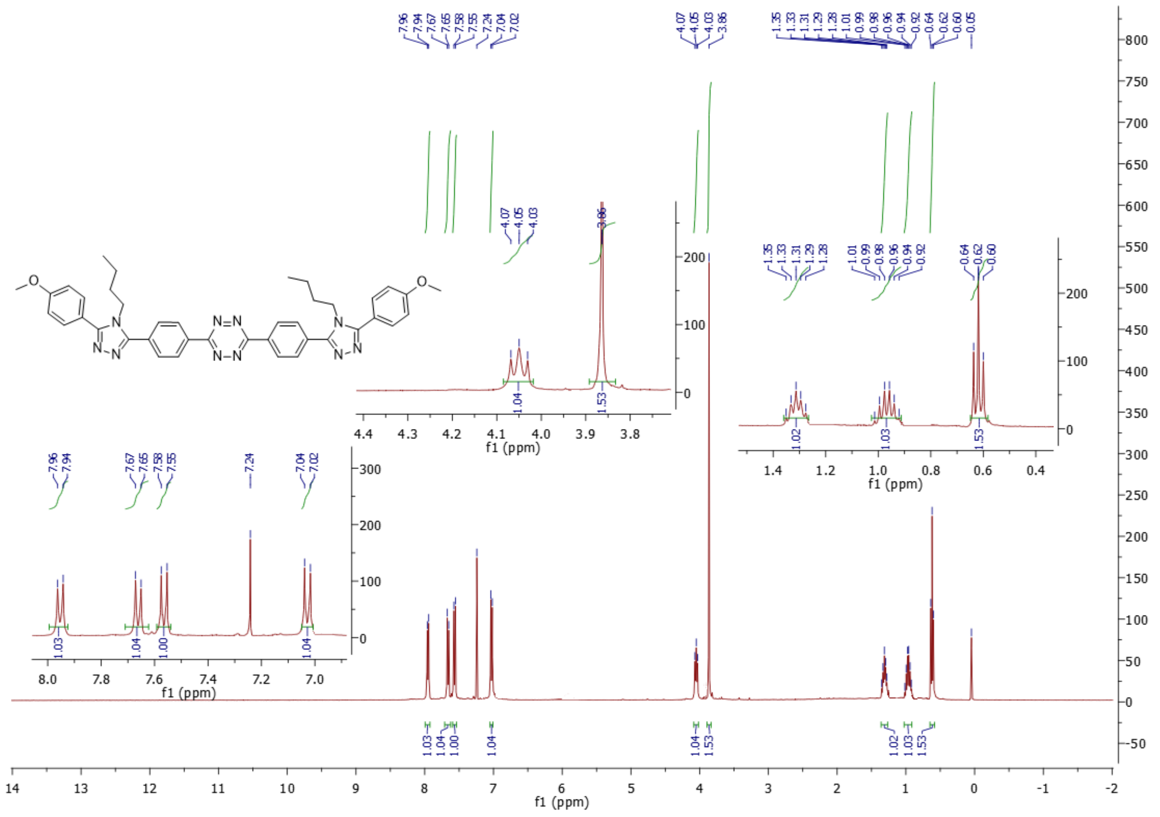

Figure S33. ^{1}H NMR spectra (400 MHz, $CDCl_3$) of 3,6-bis(4-(4-butyl-5-(4-methoxyphenyl)-4*H*-1,2,4-triazol-3-yl)phenyl)-1,2,4,5-tetrazine (**13f**)

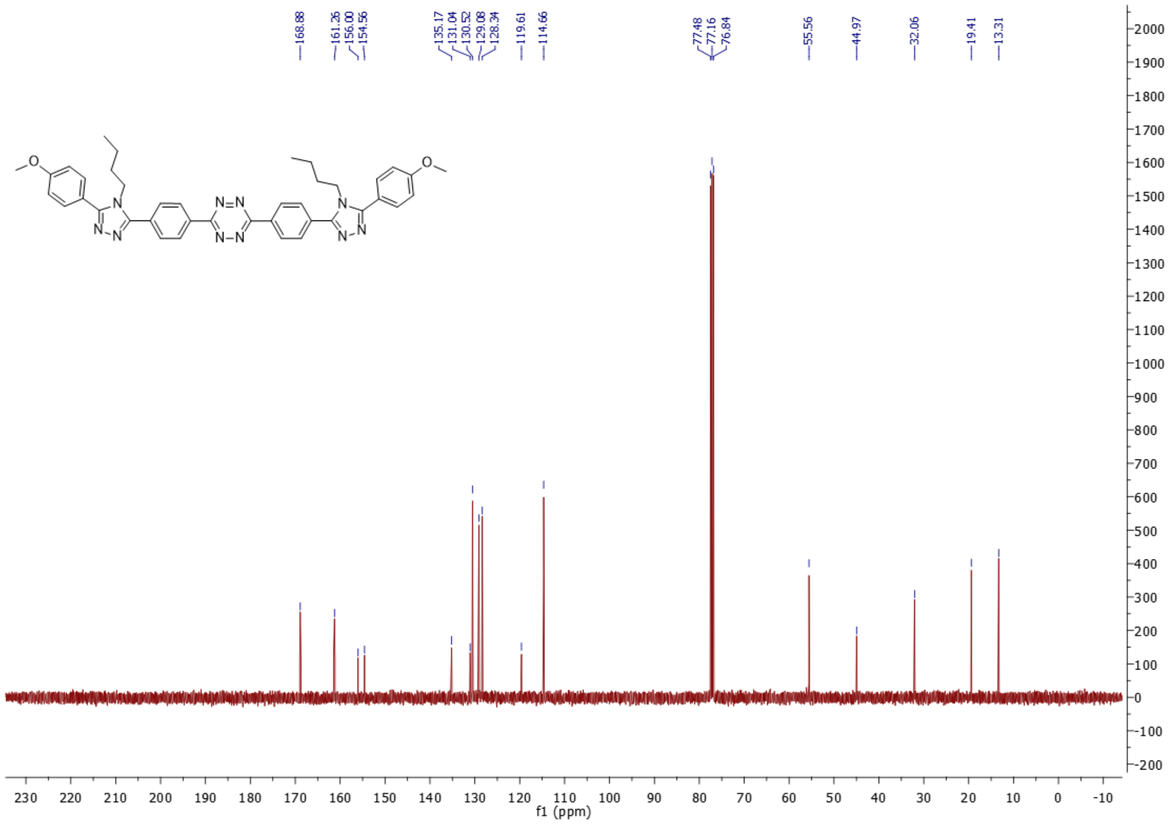

Figure S34. ^{13}C NMR spectra (100 MHz, $CDCl_3$) of 3,6-bis(4-(4-butyl-5-(4-methoxyphenyl)-4*H*-1,2,4-triazol-3-yl)phenyl)-1,2,4,5-tetrazine (**13f**)

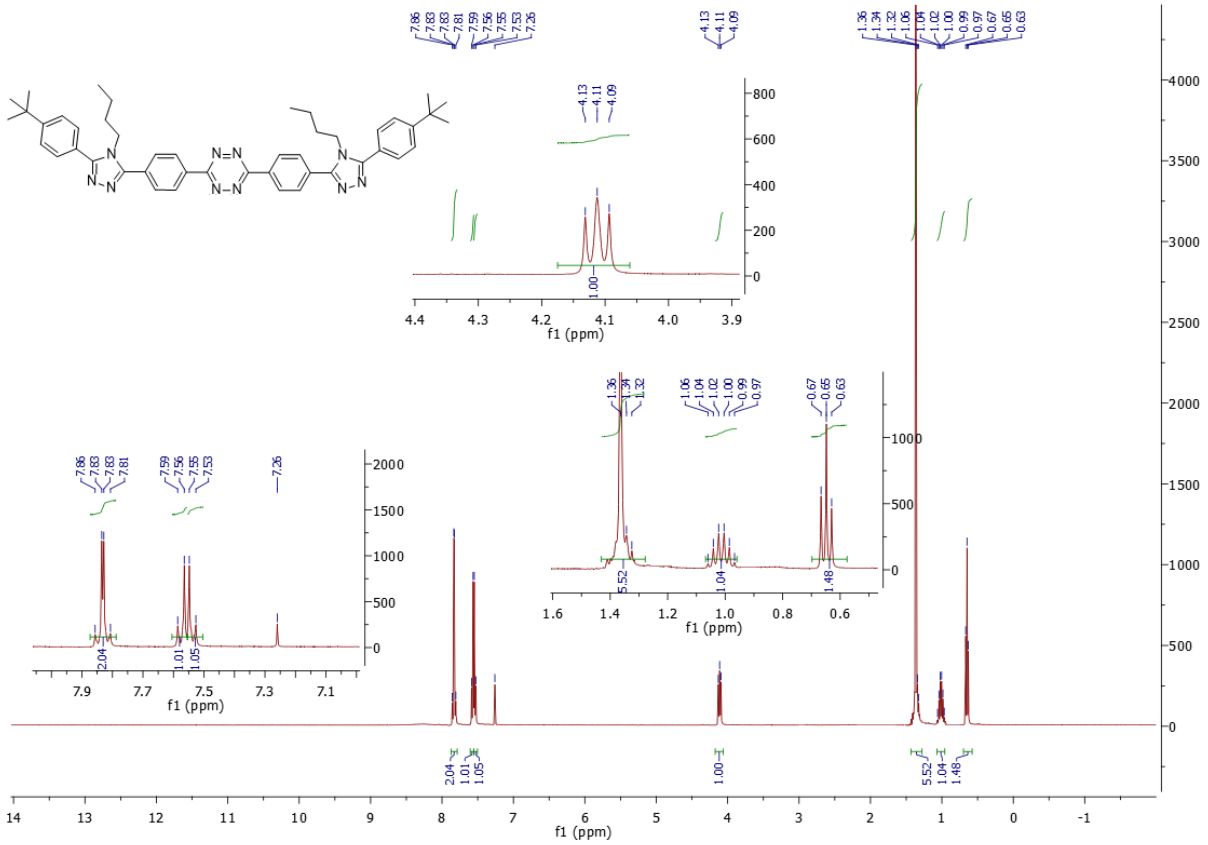

Figure S35. ^{1}H NMR spectra (400 MHz, $CDCl_3$) of 3,6-bis(4-(4-butyl-5-(4-(*tert*-butyl)phenyl)-4*H*-1,2,4-triazol-3-yl)phenyl)-1,2,4,5-tetrazine (**13g**)

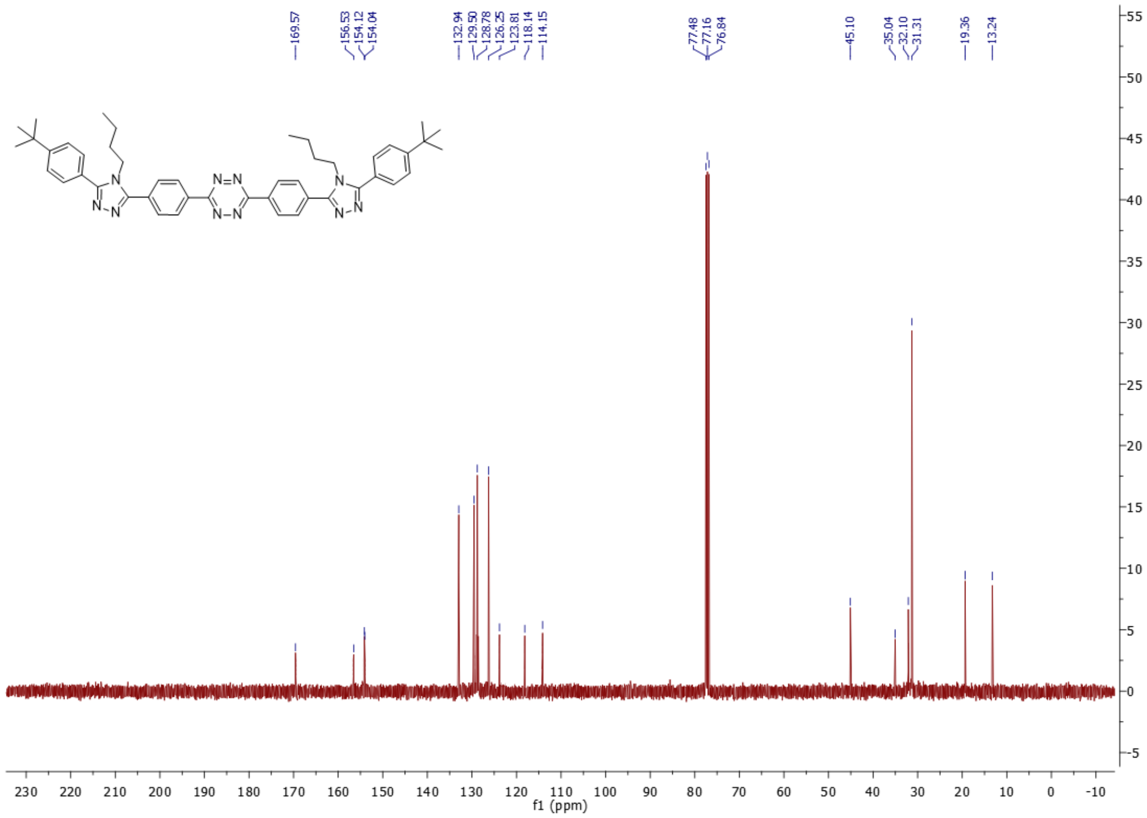

Figure S36. ^{13}C NMR spectra (100 MHz, $CDCl_3$) of 3,6-bis(4-(4-butyl-5-(4-(*tert*-butyl)phenyl)-4*H*-1,2,4-triazol-3-yl)phenyl)-1,2,4,5-tetrazine (**13g**)

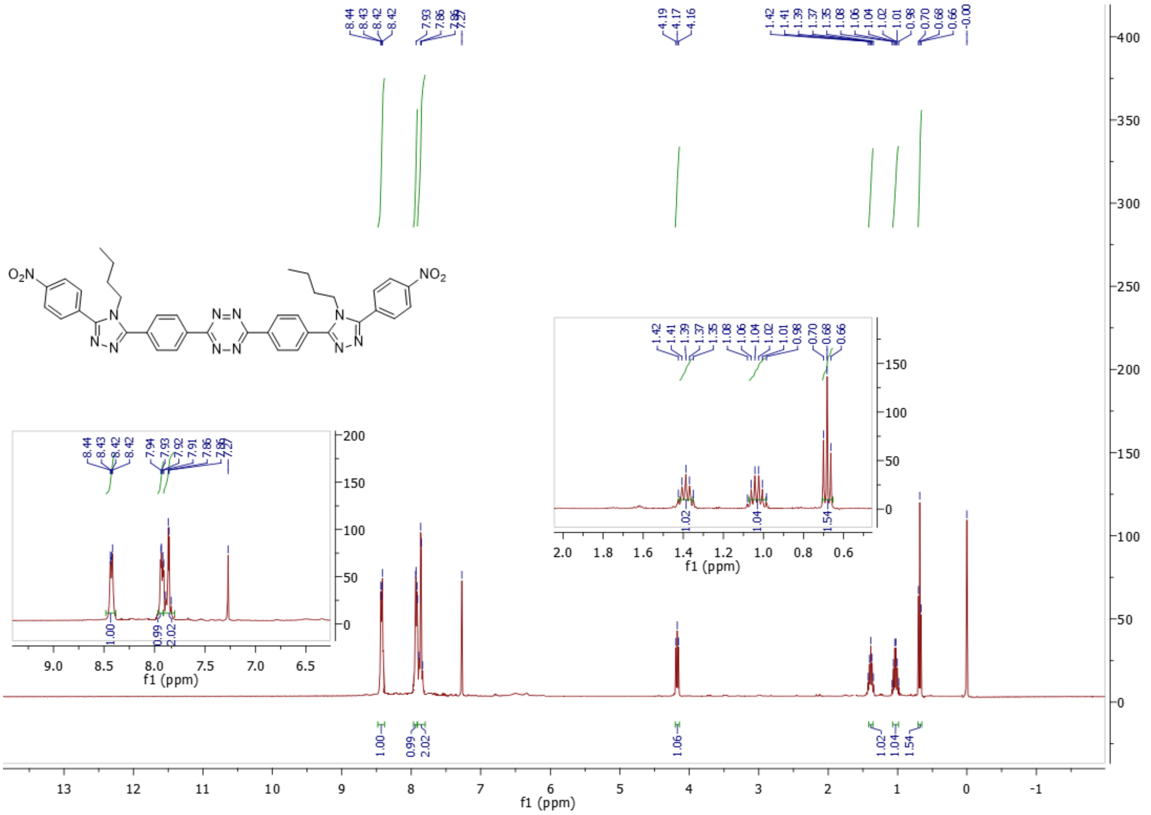

Figure S37. ^{1}H NMR spectra (400 MHz, $CDCl_3$) of 3,6-bis(4-(4-butyl-5-(4-nitrophenyl)-4*H*-1,2,4-triazol-3-yl)phenyl)-1,2,4,5-tetrazine (**13h**)

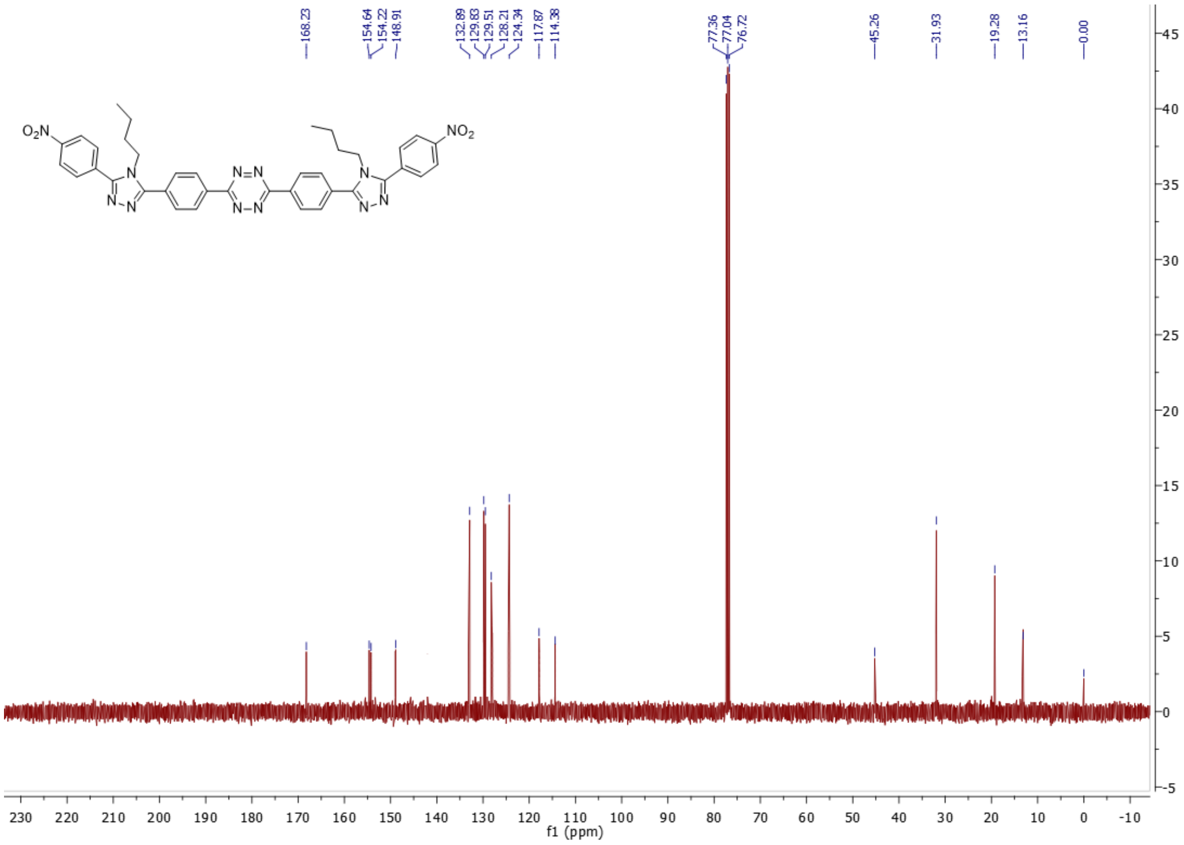

Figure S38. ^{13}C NMR spectra (100 MHz, $CDCl_3$) of 3,6-bis(4-(4-butyl-5-(4-nitrophenyl)-4*H*-1,2,4-triazol-3-yl)phenyl)-1,2,4,5-tetrazine (**13h**)

2. UV-Vis absorption spectra of compounds **13a-h**

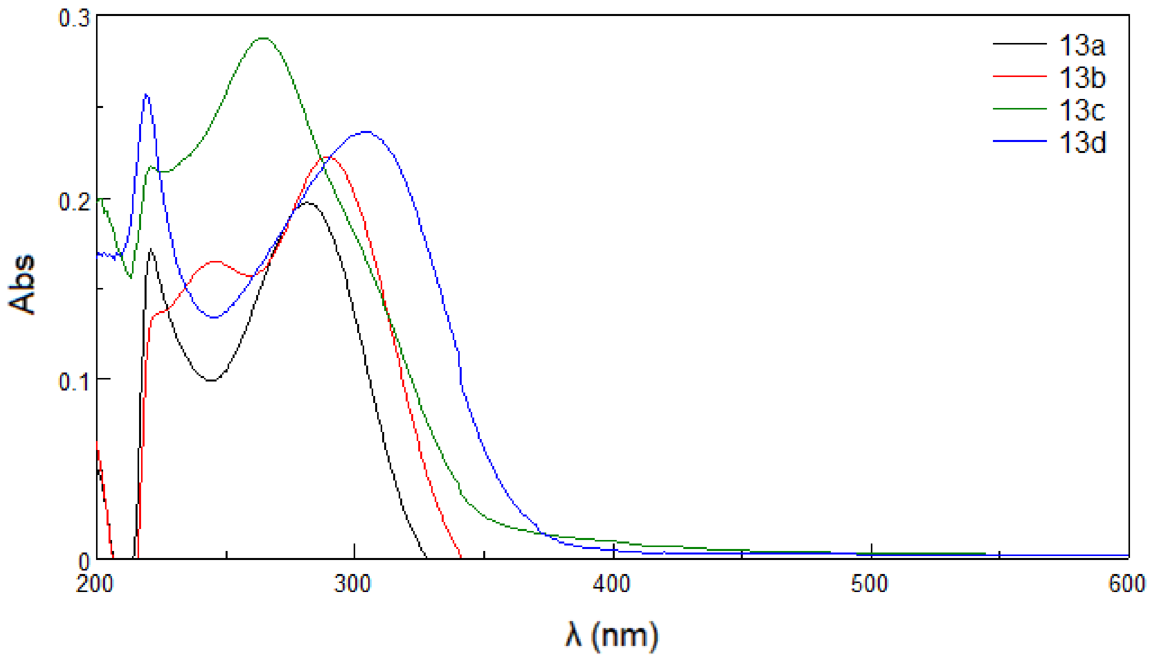

Figure S39. UV-Vis absorption spectra of compounds **13a-d**

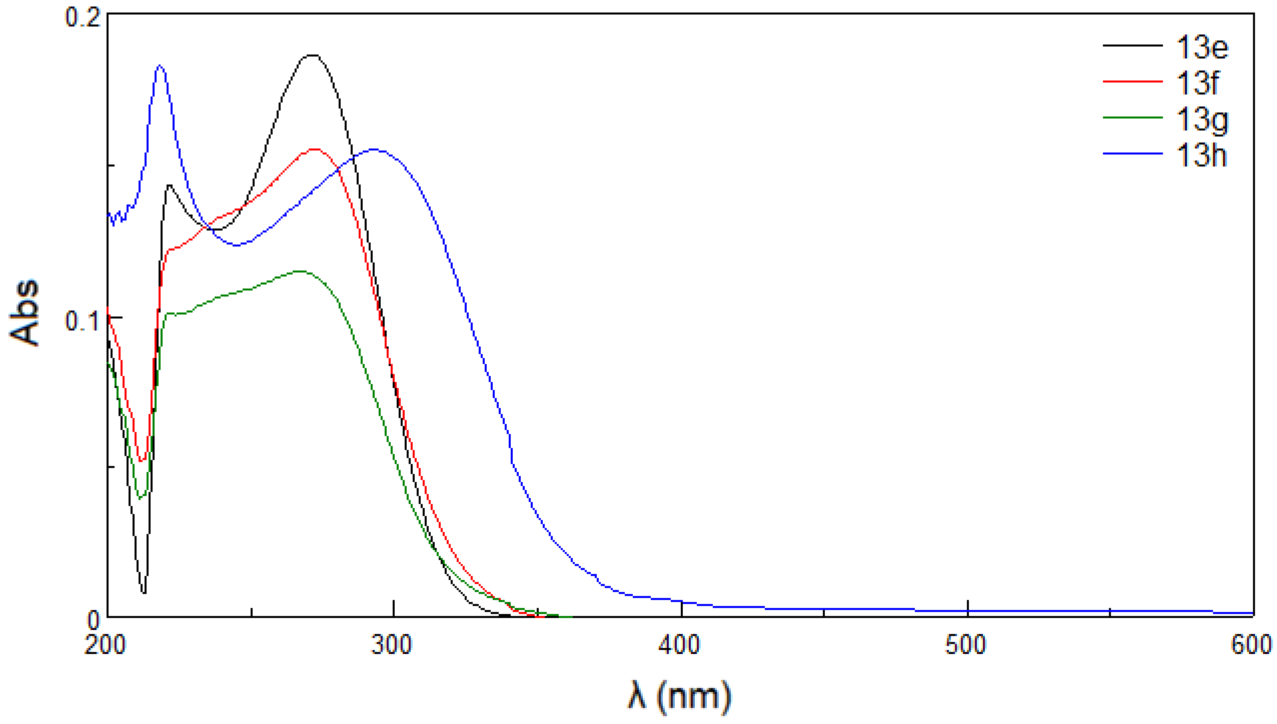

Figure S40. UV-Vis absorption spectra of compounds **13e-h**

3. 3D fluorescence spectra of compounds **13a-h** and scatter plot presenting emission data

3D fluorescence spectra of compounds **13a-h**. The color scale represents a flux of emitted photons. The number above color scales indicates the maximum relative value of emission intensity represented by color scale (and indicated in a respective figure). The unit of measurement in each spectrum represents the same number of emitted photons per second, i.e. fluorescence intensity values in all spectra can be directly compared.

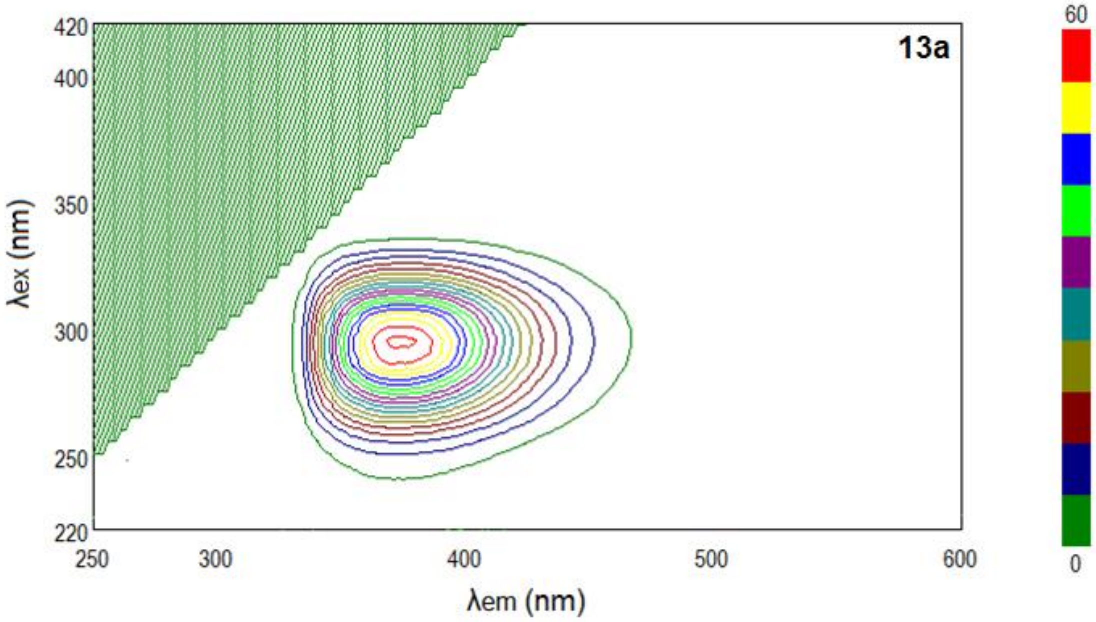

Figure S41. 3D fluorescence spectra of compound **13a**

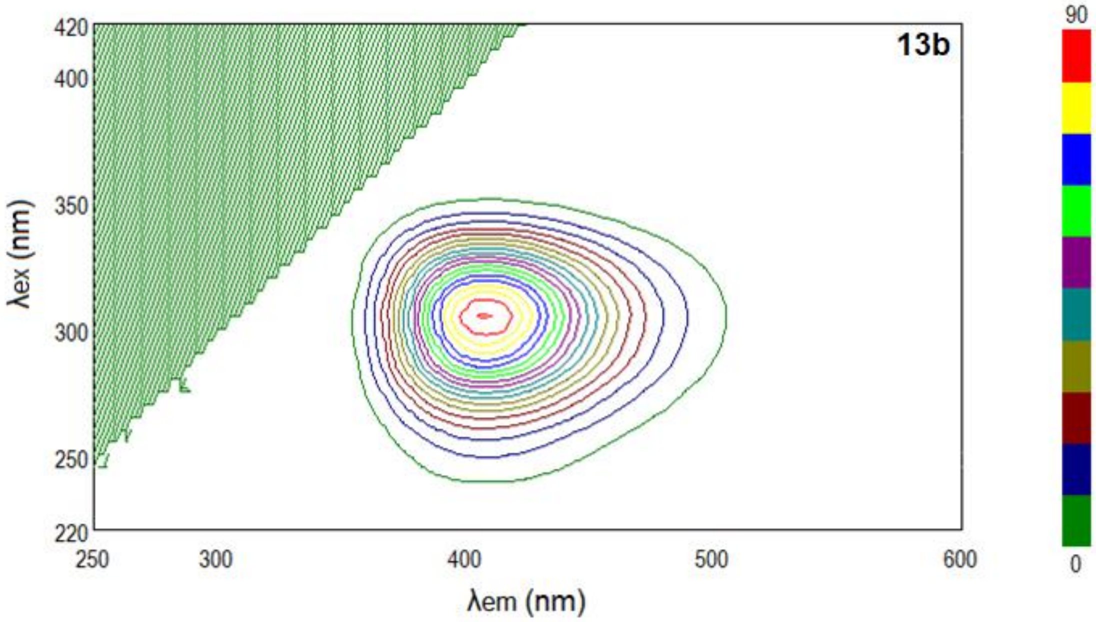

Figure S42. 3D fluorescence spectra of compound **13b**

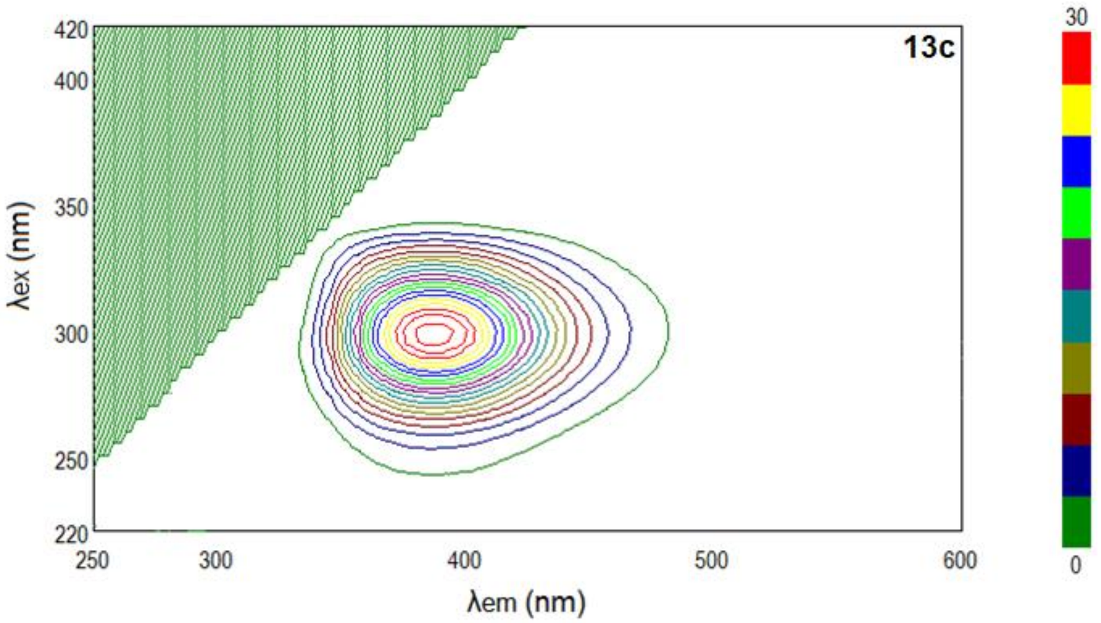

Figure S43. 3D fluorescence spectra of compound **13c**

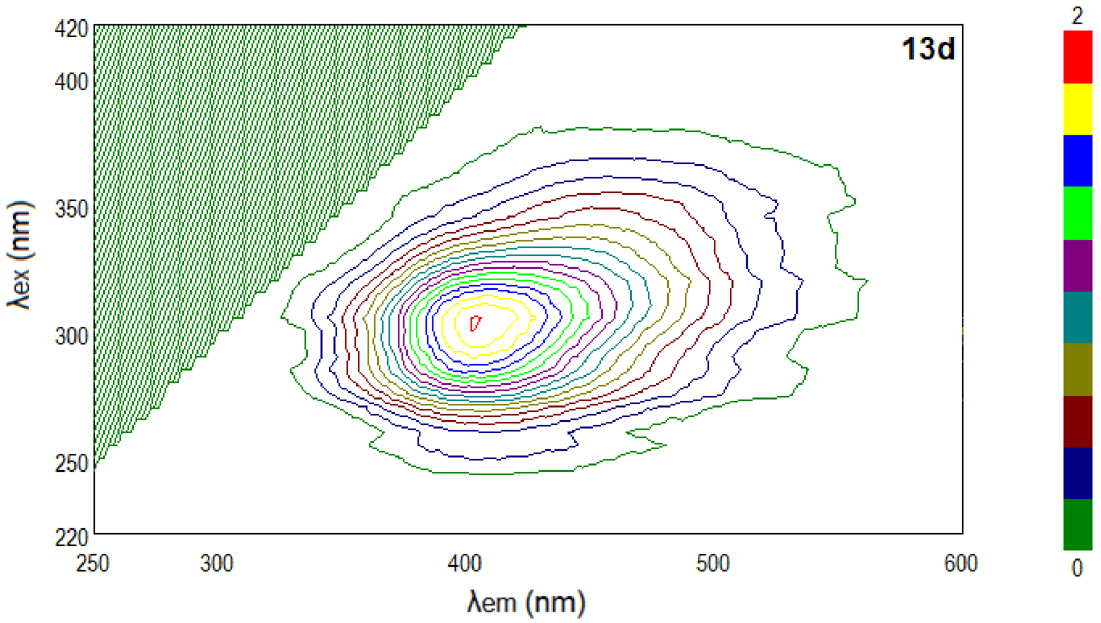

Figure S44. 3D fluorescence spectra of compound **13d**

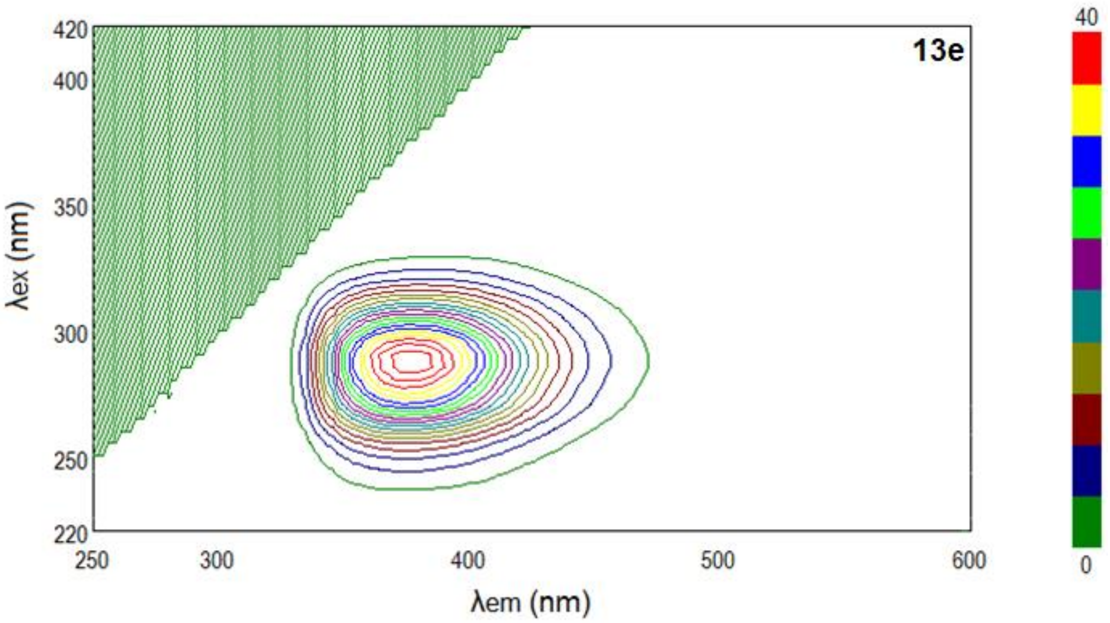

Figure S45. 3D fluorescence spectra of compound **13e**

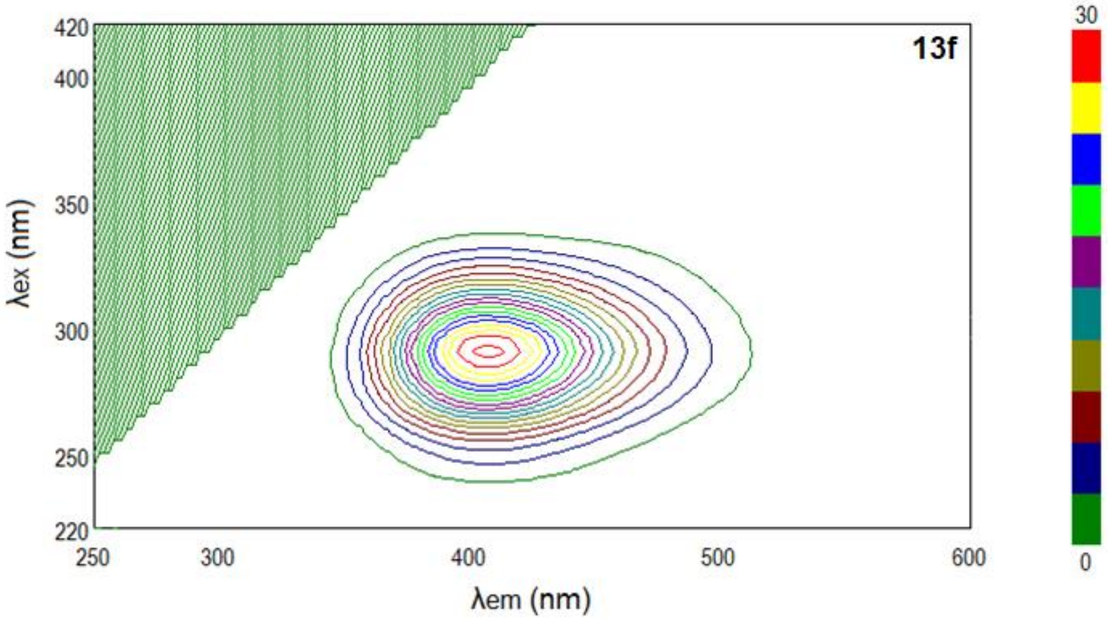

Figure S46. 3D fluorescence spectra of compound **13f**

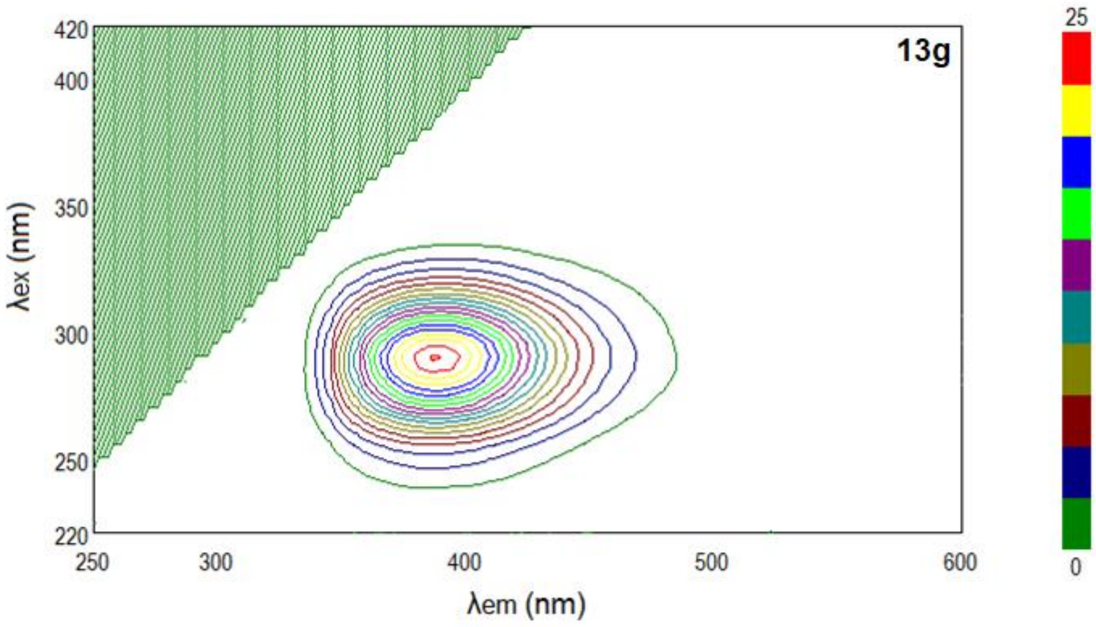

Figure S47. 3D fluorescence spectra of compound **13g**

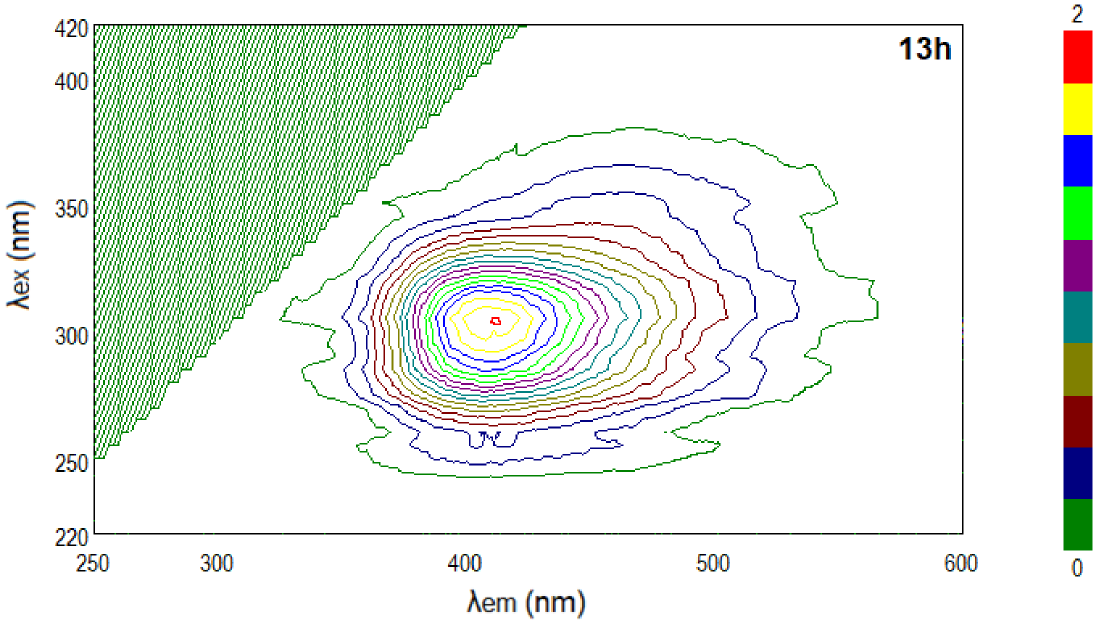

Figure S48. 3D fluorescence spectra of compound **13h**

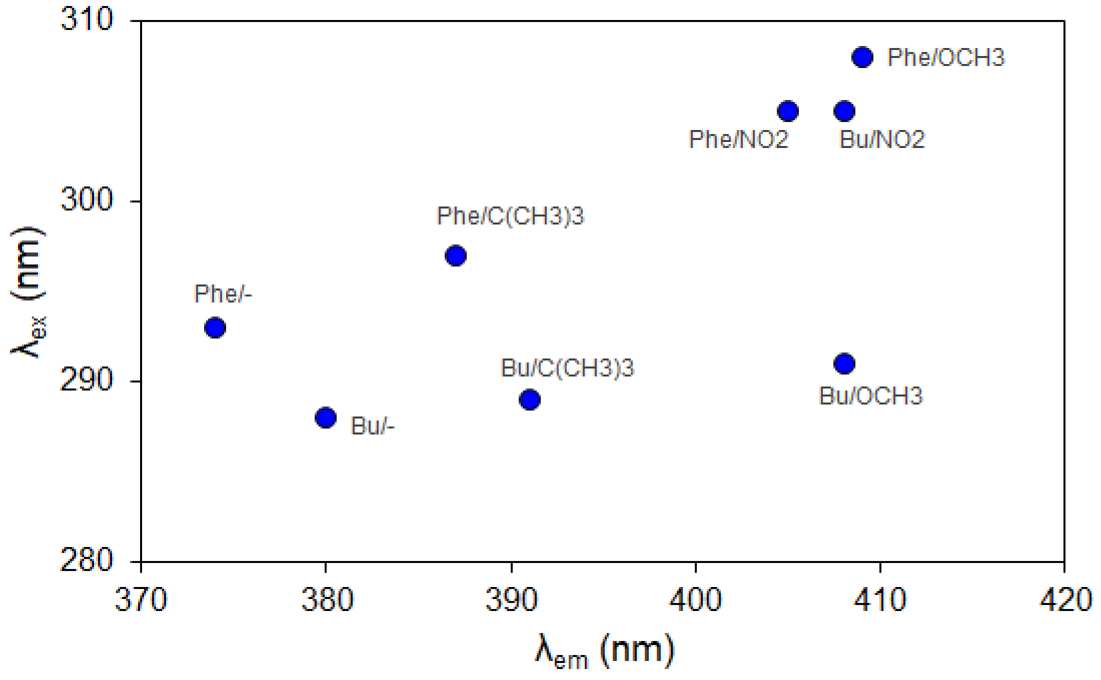

Figure S49. Position of global fluorescence maxima in **13a-h**. Labels indicate R^2/R^1 substituents in the studied compounds.
